# Latitude and protection affect decadal trends in reef trophic structure over a continental scale

**DOI:** 10.1002/ece3.6347

**Published:** 2020-06-29

**Authors:** Elizabeth M. P. Madin, Joshua S. Madin, Aaron M. T. Harmer, Neville S. Barrett, David J. Booth, M. Julian Caley, Alistair J. Cheal, Graham J. Edgar, Michael J. Emslie, Steven D. Gaines, Hugh P. A. Sweatman

**Affiliations:** ^1^ Department of Biological Sciences Macquarie University Sydney NSW Australia; ^2^ School of Life Sciences University of Technology Sydney Sydney NSW Australia; ^3^ Hawai'i Institute of Marine Biology University of Hawai'i Kane'ohe HI USA; ^4^ School of Natural and Computational Sciences Massey University Auckland New Zealand; ^5^ Institute for Marine and Antarctic Studies University of Tasmania Hobart TAS Australia; ^6^ School of Mathematical Sciences Queensland University of Technology Brisbane QLD Australia; ^7^ Australian Research Council Centre of Excellence for Mathematical and Statistical Frontiers The University of Melbourne Parkville VIC Australia; ^8^ Australian Institute of Marine Science Townsville QLD Australia; ^9^ Bren School of Environmental Science and Management University of California Santa Barbara CA USA

**Keywords:** coral reef, cross‐ecosystem, fisheries, food web, kelp forest, marine reserve

## Abstract

The relative roles of top‐down (consumer‐driven) and bottom‐up (resource‐driven) forcing in exploited marine ecosystems have been much debated. Examples from a variety of marine systems of exploitation‐induced, top‐down trophic forcing have led to a general view that human‐induced predator perturbations can disrupt entire marine food webs, yet other studies that have found no such evidence provide a counterpoint. Though evidence continues to emerge, an unresolved debate exists regarding both the relative roles of top‐down versus bottom‐up forcing and the capacity of human exploitation to instigate top‐down, community‐level effects. Using time‐series data for 104 reef communities spanning tropical to temperate Australia from 1992 to 2013, we aimed to quantify relationships among long‐term trophic group population density trends, latitude, and exploitation status over a continental‐scale biogeographic range. Specifically, we amalgamated two long‐term monitoring databases of marine community dynamics to test for significant positive or negative trends in density of each of three key trophic levels (predators, herbivores, and algae) across the entire time series at each of the 104 locations. We found that trophic control tended toward bottom‐up driven in tropical systems and top‐down driven in temperate systems. Further, alternating long‐term population trends across multiple trophic levels (a method of identifying trophic cascades), presumably due to top‐down trophic forcing, occurred in roughly fifteen percent of locations where the prerequisite significant predator trends occurred. Such alternating trophic trends were significantly more likely to occur at locations with increasing predator densities over time. Within these locations, we found a marked latitudinal gradient in the prevalence of long‐term, alternating trophic group trends, from rare in the tropics (<5% of cases) to relatively common in temperate areas (~45%). Lastly, the strongest trends in predator and algal density occurred in older no‐take marine reserves; however, exploitation status did not affect the likelihood of alternating long‐term trophic group trends occurring. Our data suggest that the type and degree of trophic forcing in this system are likely related to one or more covariates of latitude, and that ecosystem resiliency to top‐down control does not universally vary in this system based on exploitation level.

## Graphical abstract

Using time‐series data for 104 reef communities spanning tropical to temperate Australia from 1992 to 2013, we quantified relationships among long‐term trophic group population density trends, latitude, and exploitation status over a continental‐scale biogeographic range. These data suggest that the type and degree of trophic forcing in this system are likely related to one or more covariates of latitude, and that ecosystem resiliency to top‐down control does not universally vary in this system based on exploitation level.

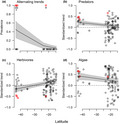

## 1 INTRODUCTION

The relative roles of top‐down (consumer‐driven) and bottom‐up (resource‐driven) forcing in exploited marine ecosystems have long been of interest to ecologists (Menge, 2000) and have remained a topic of much interest in recent years (Frank, Petrie, & Shackell, 2007; Lynam et al., 2017). This topic has become increasingly important as human activities, such as fishing and other types of harvest, have continued to alter both consumers and resources in marine systems worldwide (McCauley et al., 2015). The global loss of large predators both on land and in the sea (Estes et al., 2011; McCauley et al., 2015) has generated interest in the wider consequences for food webs of declines in predation. Despite historical controversy (Polis, 1999) and continued inquiry (Baum & Worm, 2009; Terborgh, 2015), however, no consensus has emerged regarding the relative roles of top‐down and bottom‐up forcing across different marine systems globally, how these roles vary biogeographically (but see Boyce, Frank, Worm, & Leggett, 2015; Frank et al., 2007), nor the conditions under which changes in consumer populations result in cascading population changes at lower trophic levels.

Time series of marine stock abundances have been used in recent years to diagnose mechanisms of ecosystem change, yielding valuable information on ecosystem susceptibility to top‐down trophic control and, therefore, ecosystems' resiliency to exploitation (Frank et al., 2007; Frank, Petrie, Shackell, & Choi, 2006). Specifically, these studies have examined the direction and magnitude of population trends in adjacent trophic groups (i.e., trophic groups with a consumer–resource relationship) to determine the type of trophic control driving changes observed over multiyear time scales. Top‐down control is indicated by negative correlations between consumers and their resources (e.g., predators and prey) over time for a given location (Frank et al., 2007), because all else being equal, more consumers should result in fewer prey (and vice versa) if consumption rate is the limiting factor. By contrast, bottom‐up control is indicated by positive associations between consumers and resources, because more resources should result in more consumers (and vice versa) if food availability is the limiting factor. This phenomenon is referred to as trophic forcing. Top‐down trophic forcing occurs when ecosystems are dominated by changes at the top of the food web resulting in changes at lower food‐web levels, whereas bottom‐up trophic forcing is the result of changes in lower‐level resources (e.g., primary producers) driving changes in upper‐level food‐web components. The sign (positive or negative) of the correlation coefficient between adjacent groups' population sizes over time can be used as an indication of trophic forcing (Boyce et al., 2015; Frank et al., 2006; Petrie, Frank, Shackell, & Leggett, 2009). Under this assumption, positive values indicate that two trophic groups increase or decrease over time in concert and suggest bottom‐up trophic forcing predominates; negative values indicate that trophic groups show opposing trends over time and thus suggest that top‐down trophic forcing predominates. Alternating population trends across more than two trophic groups are one (of many) potential indicators of a trophic cascade (Paine, 1980), “the time‐honored focal point of food‐web dynamics” (Polis, Sears, Huxel, Strong, & Maron, 2000:473). Community‐level trophic cascades (as distinguished from species‐level trophic cascades; Polis, 1999) are detected by alternating levels of or trends in density/biomass of entire trophic groups (rather than subsets of individual species) and significantly alter primary producer biomass distribution throughout an entire ecosystem (Polis et al., 2000). We focus here on describing population trends over time in whole trophic groups within ecosystems; therefore, our findings have relevance for when and where community‐level trophic cascades might be expected to occur. Exploited ecosystems are particularly well‐suited to this type of analysis because targeted populations are expected to decline over time when harvested at unsustainable levels. Conversely, in the case of no‐take marine reserves (hereafter “reserves”, i.e., areas protected from all extractive activities), populations of harvested species are expected to increase over time as they recover from previous exploitation.

Synthetic evidence from the literature suggests that the relative roles of top‐down and bottom‐up forcing vary biogeographically across different marine systems. For example, Frank et al. (2007) found in the North Atlantic that trophic control tended to be more bottom‐up, or resource‐driven, in higher‐latitude, higher‐diversity pelagic systems and more top‐down driven in lower‐latitude, less diverse systems. Likewise, a meta‐analysis of >50 disparate studies conducted within the northern hemisphere found that sea surface temperature was the dominant predictor of spatial variation in trophic control (Boyce et al., 2015). While these studies shed light on trophic relationships between two adjacent trophic levels, it remains unknown how these results translate when a third trophic level is considered. In this regard, the apparent discrepancy between tropical versus temperate trophic forcing is largely supported by fragmented evidence from the literature. Notable examples of community‐level trophic cascades arising from natural (non‐experimental) ecosystems, a predicted consequence of top‐down trophic forcing, have been shown from individual case studies in temperate regions (Babcock et al., 2010; Bates, Stuart‐Smith, Barrett, & Edgar, 2017; Casini et al., 2012; Daskalov, Grishin, Rodionov, & Mihneva, 2007; Estes & Duggins, 1995; Frank, Petrie, Choi, & Leggett, 2005; Myers, Baum, Shepherd, Powers, & Peterson, 2007; Steneck, Vavrinec, & Leland, 2004), yet very few (e.g., McClanahan & Muthiga, 2016) exist from tropical locations. Numerous studies have looked for, but not found, evidence of community‐level trophic cascades in tropical systems (Casey et al., 2017; Emslie et al., 2015; Sandin et al., 2008). The tropical trophic cascades that have been documented in natural ecosystems (McClanahan & Muthiga, 2016) come from systems with relatively low diversity (Worm & Tittensor, 2011) that are heavily fished (McClanahan & Muthiga, 2016). Conversely, synthetic studies covering both tropical and temperate ecosystems generally support the existence of a latitudinal gradient in trophic control (Babcock et al., 2010; Salomon et al., 2010).

Given the evidence described above, we hypothesized that within our continental‐scale study system in the southern hemisphere, lower‐diversity temperate locations would exhibit stronger top‐down trophic control than higher‐diversity tropical locations. Similarly, we hypothesized that this disparity in trophic control would result in higher likelihood of observing alternating trophic population trends across three trophic groups in temperate than tropical regions. We further hypothesized that locations with larger versus smaller changes in predator abundances over time (whether positive or negative) would be more likely to experience alternating trophic trends in lower trophic groups (i.e., herbivores; algae). Lastly, we hypothesized that no‐take marine reserves, particularly older reserves in which targeted species had experienced longer recovery periods from previous fishing, would be more likely to exhibit alternating trophic trends than locations that had been consistently open to fishing.

**FIGURE 1:**
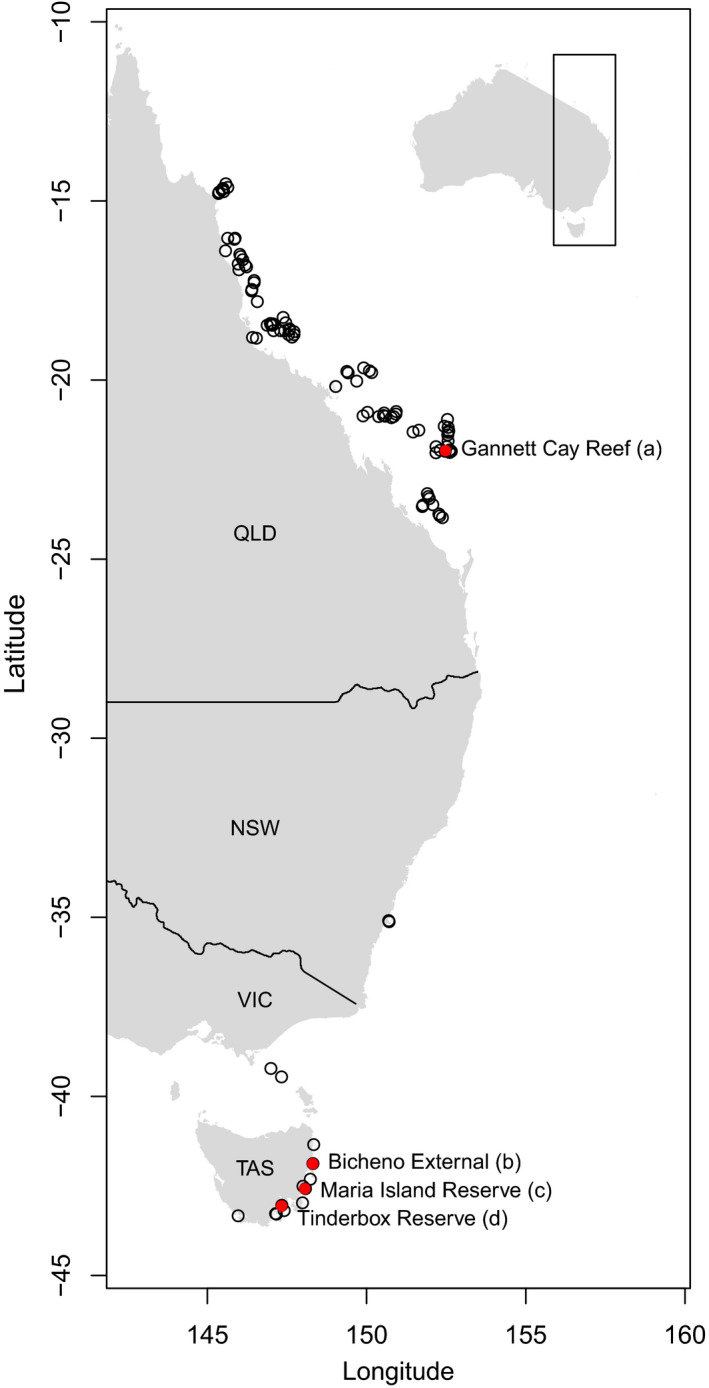
Geographic scope of the study. Sampling locations along Australia's east coast (within box on inset map). Each location contains 3–14 sites. Red locations and letters (a–d) correspond to Figure 3 where alternating long‐term density trends across multiple trophic levels were detected

To test these hypotheses, we present here long‐term (1992–2013) density trends of predators, herbivores, and benthic primary producers in coastal rocky reef and coral reef ecosystems spanning ~30 degrees of latitude along Australia's east coast. We used these data to explore temporal trends in trophic group density in the context of each other, latitude, and exploitation status. Our long‐term, large‐scale, cross‐realm comparison is broad in its spatial scope and inclusion of different ecosystem types (i.e., temperate rocky reefs to tropical coral reefs). Our data combine long‐term reef community surveys at 104 locations that collectively span tropical to temperate regions (Figure 1). Approximately half of the locations are reserves, allowing us to test for effects of reserves on targeted (predatory) and nontargeted (herbivorous) trophic groups. Our dataset includes reserves up to 34 years old, allowing detection of multitrophic responses which can take decades to emerge (Babcock et al., 2010) following release from fishing pressure. Our cross‐ecosystem approach allows tests of whether correlates of latitude may affect trophic control or the likelihood of changing consumer populations resulting in cascading density changes at lower trophic levels.

## 2 METHODS

### 2.1 Summary

We amalgamated two long‐term monitoring databases of marine community dynamics, that of the Australian Temperate Reef Collaboration (Stuart‐Smith et al., 2017) and that of the Great Barrier Reef Long‐Term Monitoring Program (Emslie et al., 2015). The combined dataset was used to test our hypotheses along the Australian east coast (Figure 1).

We looked for positive or negative trends in density of each of three key trophic levels (predators, herbivores, and algae) at each of the 104 locations across the entire time series at each site. Time series encompass the 3‐ to 20‐year survey range for each site within a location. In terms of the time‐series duration post‐reserve implementation, the majority (95%) of locations had time series spanning 9–34 years. All locations had 6 or more years of post‐reserve‐implementation survey data.

Our criterion for identifying alternating trophic trends was a statistically significant change in density of all three trophic groups over time, with adjacent trophic groups showing opposite trends (e.g., predators increase; herbivores decrease; algae increases, or vice versa).

### 2.2 Trophic groups

Benthic substrates were defined as “crustose coralline algae” (all nongeniculate coralline algae), “turf algae,” “other algae” (all other macroalgae and seagrass), “soft coral,” “hard coral,” “sponges,” “other” (e.g., other sessile invertebrates), and “abiotic.” Fish and benthic invertebrate species were categorized as either “higher carnivores,” “benthic carnivores,” “herbivores,” “planktivores,” “corallivores,” or “detritivores” using a combination of observations, expert knowledge and FishBase (www.fishbase.org). A complete list of fish and benthic invertebrate species' trophic group assignments can be found in Table S4.

Given the diet composition of these groups, and thus potential predator–prey relationships, all key ecologically relevant constituents were included in analyses and these groups varied by system type. We delineated these groupings based on the fact that there is a shift from dominance by fish herbivory to dominance by invertebrate herbivory from tropical to temperate regions, and we chose to focus our analyses on the key trophic groups and their constituents that are expected to interact (i.e., relevant predators, herbivores, and primary producers within each system). In tropical systems, *predators* consist of large‐bodied species of piscivorous fishes, *herbivores* consist of herbivorous fishes, and *algae* consist of “turf algae” and “other algae” (i.e., excluding “crustose coralline algae,” which is not generally palatable to herbivores). We only included larger‐bodied piscivore species that are known or presumed to occupy a high trophic position (Frisch et al., 2016) and that are capable of consuming herbivores. It was not necessary to apply a size filter to tropical predators because juvenile reef‐dwelling piscivores were encountered extremely rarely in censuses. We note that gape limitation of some piscivorous fish species will prevent them from preying on the largest size classes of herbivorous reef fishes (e.g., labrids (scarini)). However, the numerically dominant juveniles and subadult size classes of herbivores are vulnerable to predation by most piscivores. For example, the dietary importance of larger‐bodied herbivore families, such as Labridae (Scarini) and Acanthuridae, to a key reef fishery species (*Plectropomus leopardus*) is reflected in these herbivores' IRI (index of relative importance) rankings. Out of 22 families observed in *P. leopardus* stomach contents, they ranked 3rd and 8th, respectively (St John, Russ, Brown, & Squire, 2001). All known herbivorous fishes other than blennies (which are often cryptic) and kyphosids are thus included in the group *herbivores*. Some families, such as Pomacentridae, include both herbivores and nonherbivores; only herbivorous species in these families were included. We did not include herbivorous macroinvertebrates because that are very rare (i.e., urchins) or have been shown to exert minimal grazing impact relative to fishes in this system (e.g., *Trochus* and *Turbo* spp.; pagurid crabs; Klumpp & Pulfrich, 1989). Because of the disproportionate representation of the largely herbivorous family Pomacentridae (damselfishes) in some piscivorous fishes' diet (e.g., St John et al., 2001), we reran our analyses using only this family in the *herbivore* trophic group. This analysis gave qualitatively similar results, so we did not limit the *herbivore* trophic group to this particular family. In warm temperate and cool temperate systems, *predators* consist of large‐bodied carnivorous (benthic invertivorous) fishes (>30 cm TL) and invertebrates (i.e., lobsters > 10 cm carapace length), *herbivores* consist of herbivorous invertebrates (i.e., urchins, abalone, and genus *Turbo*), and *algae* consist of “other algae” (specifically, canopy‐forming brown algae and foliose brown, green, and red algae). The *predator* group represents those species that are capable of handling and consuming prey that are physically defended (e.g., by spines/shells).

### 2.3 Exploitation status

We started each trend analysis at the year the reserve was implemented for reserve locations. To control for confounding temporal (e.g., environmental) factors, we used the same starting year for trends at fished locations in the same geographical area. We divided exploitation into four categories: “early reserve” (locations in which our monitoring data began at the time of reserve implementation; *N* = 36); “late reserve” (reserve locations where surveys began 11–16 years after reserve implementation; *N* = 15); “always fished” (fished locations that were fished prior to the start of surveys and continue to be fished; *N* = 50); and “newly fished” (locations that were reserves immediately prior to the start of surveys but were opened to fishing at the time the surveys began; *N* = 3).

### 2.4 Data synthesis

Warm temperate and cool temperate surveys, covering New South Wales (NSW) and Tasmania (TAS), respectively, included densities for fish and nonsessile benthic invertebrates and percentage cover of benthic substrates. All observable fish species were included in these surveys, and both fishes and invertebrates were identified to the species level. Tropical surveys, covering Queensland (QLD)'s Great Barrier Reef, included densities for fish only and percentage cover of sessile benthic organisms and benthic substrates. Species from the ten most common fish families (215 species in total) were included in these surveys. These surveys did not count tropical apex predators with low occurrence and wide home ranges (e.g., sharks; jacks [family: Carangidae]) due to the unreliable estimates that noninstantaneous diver surveys often produce for these species (Ward‐Paige, Flemming, & Lotze, 2010). Details of warm/cool temperate survey methods can be found in Barrett, Edgar, Buxton, and Haddon (2007) and Barrett, Buxton, and Edgar (2009), and tropical survey methods are described in Emslie et al. (2015) and on the AIMS website. Differences in in situ survey methods are accounted for by the fact that all trophic group time‐series trends were constructed from data at a single location. Subsequent analyses use these standardized trends, which is assumed to be a “common currency,” to compare among locations, regions, and exploitation statuses.

Benthic cover was measured differently in temperate and tropical datasets. Temperate surveys (from states “NSW” and “TAS” in Figure 1) used a three‐dimensional measure of cover recorded from the benthos to the canopy and could therefore exceed 100% cover. Tropical datasets (from state “QLD” in Figure 1) include a two‐dimensional measure of cover and cannot exceed 100% cover. As comparisons are made only within locations and are therefore relative, we did not cap temperate cover at 100%.

Data were structured hierarchically with sites nested within locations (N locations = 104). Tropical locations were typically individual reefs or reefs surrounding islands with three sites surveyed at each reef. Temperate locations were typically sections of mainland or island coastline with three‐14 sites within a location. Density and percent cover data for each trophic group were aggregated by first summing the totals of each group within each transect and then calculating the mean across transects and sites within a location for each year of data collection. Site was used as the unit of replication because individual transects were contiguous at warm and cool temperate sites and were thus not independent.

### 2.5 Trophic group trend detection

Because density metrics differed among monitoring programs and trophic grouping, we standardized density data for each trophic group time series (predators, herbivores, and algae) at each of the 104 locations (i.e., rescaled with a mean of zero and standard deviation of one). All of the 312 trophic group time series were between three and 19 yearly time‐points long, and all were made up of no fewer than twelve surveys (i.e., independent surveys in a unique combination of year by location by site‐within‐location; Figure S1). For each location’s time series, we calculated Pearson's correlation coefficients and fitted linear regression models, the latter to determine the significance of trends. We also fitted regression models with an auto‐regressive factor (AR‐1), which weights residuals for one year as a function of the previous year (Zuur, Ieno, Walker, Saveliev, & Smith, 2009) and is therefore conservative. The AR‐1 correlation structure requires unique values within years, and we thus nested sites within year; however, this structure meant that we could only apply the AR‐1 model to locations with relatively even site‐level sampling across all years within time series. The two regression approaches were overwhelmingly in agreement, and the two instances in which they were not did not affect the overall results. Therefore, we only present results without the auto‐regressive term in Table S3.

### 2.6 Trend analysis

There were 24 locations with significant changes in predator densities through time (16 increases; eight decreases; Table S1). A significant positive or negative change in predator density was the prerequisite for detecting top‐down, alternating trophic trends over all three tropic groups. This is because only with significant changes in the top trophic group would lower trophic groups be expected to respond if top‐down control was operating. Of these 24 locations, four of the increasing predator trends also showed significant trends for both lower trophic groups (i.e., herbivores and algae). To determine whether the alternating trophic trends within these 24 locations were associated with latitude or exploitation status (early reserve, late reserve, always fished, and newly fished), we used a generalized linear model with a binomial response variable (0: no alternating trophic trends associated with significant predator trend; 1: alternating trophic trends associated with significant predator trend) and the logit link function. For model selection, we followed the procedure recommended by Zuur et al. (2009) of backward stepwise removal of nonsignificant model terms (*p* > .05) from the full model (incorporating both latitude and exploitation status as explanatory variables) based on AIC (Table S2). Statistical outputs are given in Table S3; model estimates with standard errors are presented in Figures 5A and 6A.

To determine whether trends in trophic groups were associated with latitude or exploitation status, we used weighted linear regression models. Correlation coefficients are sensitive to sample size and are also bounded by −1 and 1. In line with established meta‐analytic methods, we converted coefficients to Fisher's *z* values and weighted the regression models by the inverse of the variance of *z* (Borenstein, Hedges, Higgins, & Rothstein, 2009). Model selection procedures were performed as above (Table S2). Statistical outputs are given in Table S3; model fits and confidence intervals were converted back to correlation coefficients for presentation in Figures 3b–d and 4b–d.

## 3 RESULTS

**FIGURE 2:**
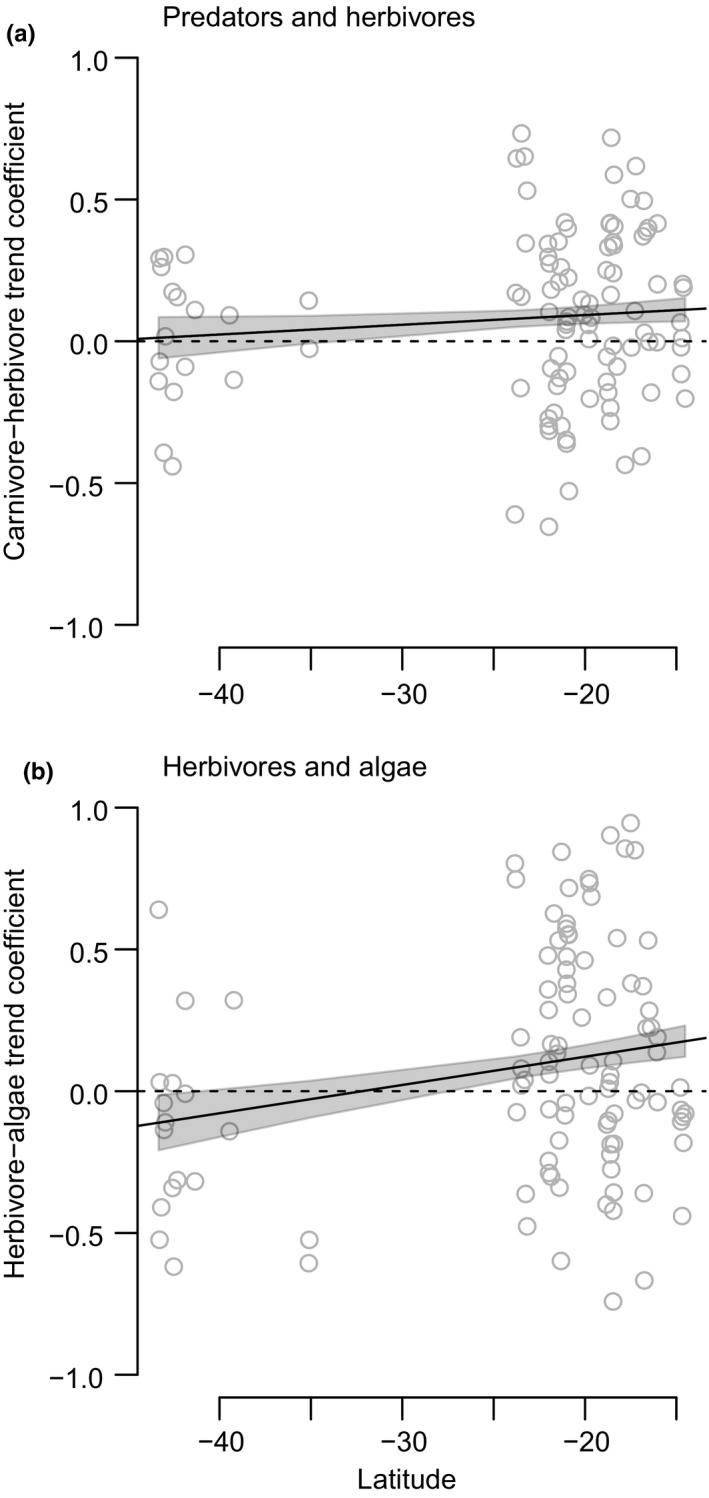
Relationship between latitude and the type of trophic forcing detected for trophic group pairs. In (a) and (b), points represent the Pearson's product moment correlation coefficient between the two named trophic groups' time‐series trends at each of the study's 104 locations. Positive values indicate that the two trophic groups increased or decreased over time in concert; negative values indicate that the groups showed opposing trends; and overlap with dashed line (0) indicates no association. Shaded areas are standard errors of model parameters. In both (a) and (b), trophic group pairs' trends were more likely to be positively related in tropical than temperate areas. Predator and herbivore trend coefficients showed no consistent association in temperate areas (a), and herbivore and algae trend coefficients showed an overall negative association in temperate areas (b). Positive associations between adjacent trophic groups are consistent with bottom‐up trophic forcing, whereas negative associations between adjacent trophic groups are consistent with top‐down trophic forcing. In both panels, points are jittered to improve readability

We found that trophic control in tropical systems tended on average to be bottom‐up (i.e., mean correlation coefficient between adjacent groups was positive), whereas temperate systems tended to exhibit top‐down forcing between herbivores and algae (i.e., mean correlation coefficient between adjacent groups was negative) and showed no clear association between predators and herbivores (Figure 2). Overall, herbivore–algae time‐series coefficients varied significantly with latitude (*R* ^2^ = .0373, *F*(1, 102) = 4.993, *p* = .028), but predator–herbivore coefficients did not (*R* ^2^ = .0005, *F*(1, 102) = 1.052, *p* = .3075).

**FIGURE 3:**
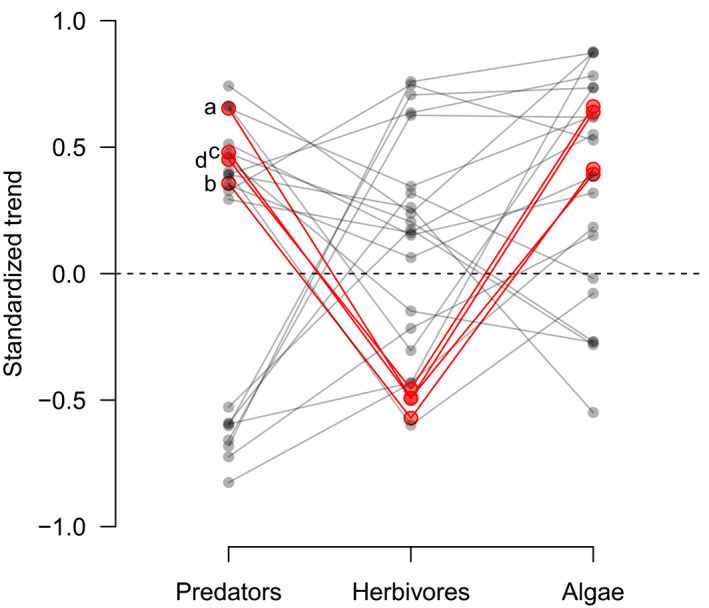
Prevalence of alternating long‐term density trends across multiple trophic levels consistent with top‐down trophic forcing. Of the 24 locations with significant positive or negative predator trends (a prerequisite for top‐down trophic forcing, when measured by significant long‐term trends), four had significant alternating trends in herbivores and algae that indicate community‐level, top‐down trophic forcing (red points and lines). Letters a–d correspond with Figures 1 and 4, 5, 6

**FIGURE 4:**
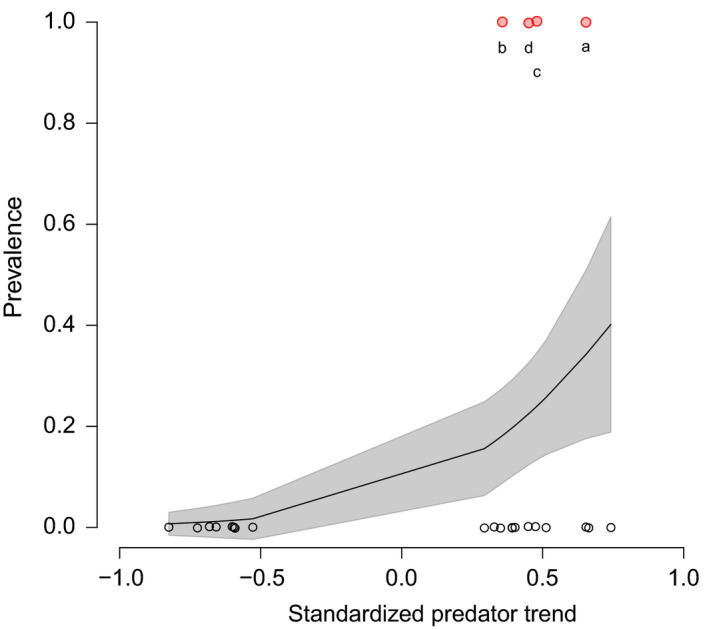
Relationship between long‐term predator density trends and prevalence of alternating long‐term density trends across multiple trophic levels. Points represent the 24 study locations with significant positive or negative predator trends. Alternating trophic group trend prevalence is shown as a function of standardized predator trends. Standard error (shaded area) visually indicates significance of differences in prevalence over predator trend strength. Points are jittered to improve readability. Red locations and letters (a–d) correspond to locations in Figures 1 and 3 where alternating long‐term density trends across multiple trophic levels were detected

We found also that alternating long‐term density trends across multiple trophic levels (a method of identifying trophic cascades), presumably due to top‐down trophic forcing, occurred in a subset of locations where significant increasing or decreasing predator trends occurred (Figure 3). Specifically, we observed 24 significant changes in predator density over time, approximately five times more than expected by chance at *α* = .05 (Figure 3; Table S1). Approximately half of these locations had 16+ years of data (minimum = 9 years), some with up to 34 years of reserve protection (Table S1). Within these 24 locations where the prerequisite significant predator trends occurred, we observed alternating long‐term density trends across multiple trophic levels in roughly 17% (four) of these locations (Figure 3), each of which showed a positive predator trend over time (Figure 4). These locations were Maria Island Reserve, Tinderbox Reserve, and Bicheno External, as well as Gannett Cay (Figure 1 and Figure S1). Maria Island, Tinderbox, and Gannett Cay are reserves in which predators increased, herbivores decreased, and algal density increased over time (Figure 3). Bicheno is fished, yet showed the same pattern, although this pattern may be partly related to multitrophic level harvest (i.e., including herbivores) rather than solely effects of predation. We found no cases of alternating trophic trends associated with significant predator declines. Four additional locations showed opposing trends between predators and herbivores (Table S1). Other locations with significant changes in predator densities showed no alternating trends (Figure 3; Table S1; Figure S1).

**FIGURE 5:**
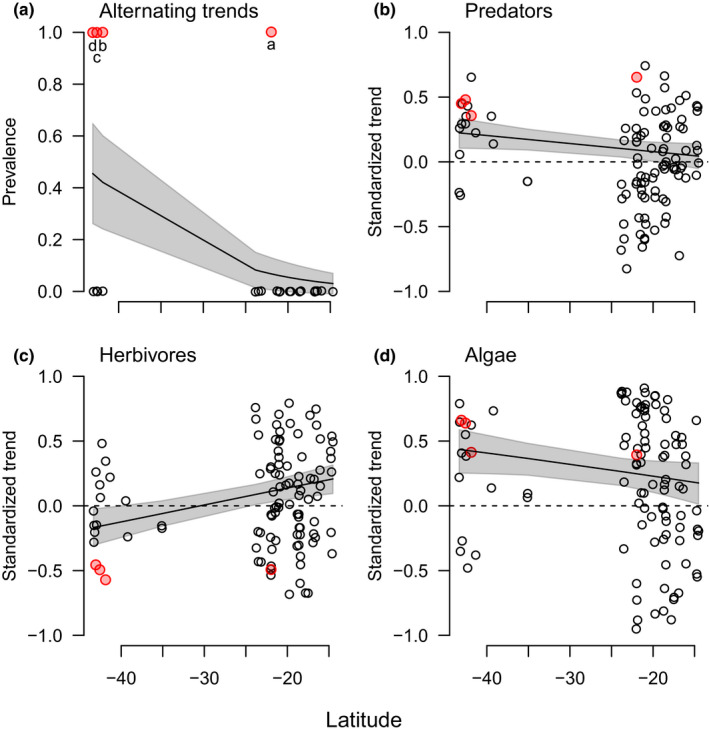
The effect of latitude. Alternating long‐term density trends across multiple trophic levels were related to latitude (A), where they were significantly more common at temperate locations. Along the x‐axis, temperate areas are towards the left and tropical areas towards the right, given that the equator is at 0 degrees latitude. There were weak latitudinal patterns associated with individual trophic group trends (B–D), with the exception of herbivores, where temperate and tropical locations showed opposite trend signs on average. Standard error (shaded area) is shown in A to visually indicate significance differences with latitude. Confidence intervals (shaded areas) are given in B–D to show where overall trends for a group are significantly different to zero. Red locations and letters (a–d) correspond to locations in Figures 1 and 3 where alternating long‐term trends across multiple trophic levels were detected

Of the 24 locations described above where the prerequisite significant predator trends occurred, the observed alternating long‐term population trends were more prevalent in temperate than tropical systems. Specifically, we observed this pattern in three (of seven) temperate study sites and one (of 17) tropical study sites (Figures 1 and 5A). We found a significant relationship between latitude and the probability of alternating trophic trends from rare in the tropics (<5% of the 17 tropical cases with significant predator trends) to relatively common in temperate areas (~43% of the 7 temperate cases with significant predator trends; Figure 5A; Table S3); however, this relationship necessarily has low statistical power due to the low number of significant predator trends—a prerequisite for detecting top‐down trophic forcing. Likewise, wholesale latitudinal trends of individual trophic groups varied significantly over latitude (Table S3), where divergences from no trend were on average greater in temperate locations (Figure 5B–D; Table S3).

**FIGURE 6:**
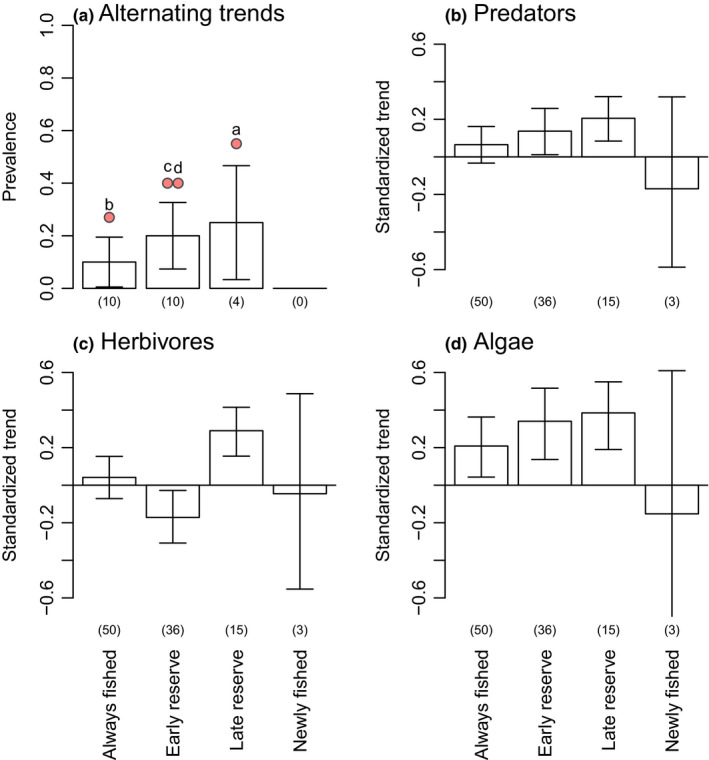
The effect of exploitation status. No significant difference was evident in the probability of alternating long‐term density trends among levels of protection from fishing (A). Protection levels span locations that have been fished since before monitoring began (“always fished”), reserves 6–20 years old (“early reserves”), older reserves (29–34 years old) for which monitoring started 11–16 years after implementation (“late reserves”), and the few reserves that were opened to fishing at the time that monitoring began (“newly fished”). Positive predator trends tended to occur in reserves (B), particularly in late reserves, and negative predator trends in newly fished areas, although the latter is not statistically different from zero (no trend). Herbivore trends were idiosyncratic (C). Algal trends were positive across the board (D) other than in newly fished areas. Numbers in parentheses are sample sizes (numbers of locations). Standard error bars are given in A to visually indicate significance differences among exploitation statuses. 95% confidence intervals are given in B–D to show where overall trends for a group differ significantly from zero (no trend). Red locations and letters (a–d) correspond locations in Figures 1 and 3 where alternating long‐term trends across multiple trophic levels were detected

Both predators and algae were more likely to exhibit the expected positive trends over time in older versus younger reserves (i.e., the slope of the density trend over time is significantly different from 0, which indicates no trend; Figure 6B,D); however, alternating long‐term density trends across multiple trophic levels were not related to exploitation status (Figure 6A).

## 4 DISCUSSION

### 4.1 Interpretation of results

We found that trophic control in tropical systems tended on average to be bottom‐up, while temperate systems tended to exhibit top‐down forcing between herbivores and algae and showed no clear association between predators and herbivores (Figure 2). This finding is largely in agreement with a previous study in pelagic North Atlantic ecosystems with similar assumptions underlying their assessment of trophic forcing over latitude (Frank et al., 2007). That study found that bottom‐up (resource‐driven) trophic control tended to dominate in higher‐latitude, higher‐diversity systems and top‐down trophic control dominated in lower‐latitude, less diverse systems. Likewise, Boyce et al.'s (2015) synthetic analysis of 52 studies in northern hemisphere marine systems found that temperature was the dominant determinant of trophic control and operated through both direct and indirect mechanisms.

Figure 3 demonstrates that significant increases or decreases in predator assemblages did not consistently result in alternating trophic group trends at lower tropic levels. Interestingly, alternating trends across multiple trophic groups were significantly more likely to occur at locations with positive (i.e., increasing), rather than negative, predator trends over time (Figure 4).

Our finding that the probability of alternating trophic trends is lower in the tropics than in temperate areas (Figure 5A; Table S3), based on a subset of the dataset, supports the notion derived from the full dataset (Figure 2) that tropical locations within our study system are less likely than temperate locations to be governed by top‐down trophic forcing. Empirical (Babcock et al., 2010; Frank et al., 2007) and synthetic (Salomon et al., 2010) studies collectively suggest that alternating trends among adjacent trophic groups may be rarer in more species‐rich, tropical systems due to greater functional redundancy within trophic groups. Our finding from a single latitudinal gradient supports the hypothesized general latitudinal gradient in the prevalence of top‐down food‐web effects based on these previous results.

Predators are well known to exhibit increases in density and/or biomass following protection from fishing (Babcock et al., 2010; Barrett et al., 2009; Edgar, Barrett, & Stuart‐Smith, 2009; Emslie et al., 2015; Russ et al., 2008), and, in some cases, predator increases have been shown to scale with reserve age (Edgar et al., 2014; Espinoza, Cappo, Heupel, Tobin, & Simpfendorfer, 2014; Friedlander et al., 2017). It is therefore unsurprising that we observed more strongly positive long‐term density trends in predator populations within older versus younger reserves (Figure 6B). These results suggest that reserves, and particularly older reserves, can restore exploited predator populations once fishing has ceased, and that this effect magnifies with time. Within this context, algal density trends are expected to be more strongly positive in older versus younger reserves (Figure 6D) if top‐down trophic forcing occurs, as suggested by Figure 4. However, herbivore trends were idiosyncratic with relation to exploitation status (Figure 6c), casting doubt on the mechanism of alternating trophic trends as the cause of this algal density trend pattern (Figure 6D) in our study system.

Contrary to our expectation, the probability of alternating long‐term population trends was statistically independent of exploitation status, although three of the four instances of this pattern did occur in reserves (Figure 4a). This latter result may be due in part to the relatively low human density on Australia's coast. As a result, Australia experiences lower overall fishing pressure than most other coastal nations (Halpern et al., 2008) despite intense pressure on a subset of species. The resultant overall light to moderate fishing pressure provides limited scope for recovery of all but the most heavily targeted species within reserves and may leave harvested trophic groups functionally intact. Specifically, under moderate fishing pressure where only a subset of predator species is targeted, numerical compensation within the predator guild may be expected to obscure community‐wide reserve effects even in lower‐diversity systems.

### 4.2 Potential mechanisms underlying observed patterns

We are aware of no a priori reason to expect that regional differences in taxa that comprise our trophic groups would alone lead to the patterns we observed. While we cannot rule out this as an explanation for our findings, and indeed it is not mutually exclusive with nontaxonomic latitudinal effects, the lack of an a priori prediction suggests that other correlates of latitude merit consideration as possible explanations. Many hypotheses have been proposed in the literature to explain disparities observed among locations in the frequency and magnitude of top‐down effects. The key classes of mechanisms cited in the literature, four of which are likely to covary with latitude, are discussed below. Disentangling the importance of these potential mechanisms is important, yet the geographic scope of our dataset (i.e., one large, but unreplicated, latitudinal gradient) does not allow us to distinguish between them. It is well known that latitude is generally correlated with both temperature and species diversity in both terrestrial (Pianka, 1966) and marine systems (Rex et al., 2002; Rohde, 1992; Roy, Jablonski, Valentine, & Rosenberg, 1998). Based on this fact combined with results from previous studies (described below) and the characteristics of our study system, we infer that the most parsimonious (and nonmutually exclusive) explanations for our findings are species diversity and resultant functional redundancy, temperature and seasonality, and/or the nature of regional fisheries and/or herbivore guilds.

Species diversity, and resultant trophic complexity, has been one of the most frequently cited mechanisms affecting top‐down control (Pace, Cole, Carpenter, & Kitchell, 1999; Paine, 1966; Polis et al., 2000; Polis & Strong, 1996; Salomon et al., 2010; Strong, 1992). The underlying logic is that more speciose systems, with their often diffuse trophic linkages and frequently higher level of omnivory, may be buffered from the effects of removal of one species or functional group by redundancies in the food web (Strong, 1992). The resulting differences in functional redundancy within trophic groups in tropical versus temperate locations—in which multiple species contribute similarly to an ecological function—may limit top‐down trophic forcing in the more speciose tropics. The geographic endpoints of our study system differ dramatically in species richness in terms of both total species richness (Tittensor et al., 2010) and richness of specific taxa such as coastal fishes, corals, and seagrasses (Edgar et al., 2017). As suggested by previous work (Emslie et al., 2015), counteracting species' density trends within trophic groups means that changes in one or more species within a trophic group rarely cause wholesale abundance changes within the next trophic level in tropical locations. This phenomenon also suggests that such effects will only be detectable if most or all species within each trophic group, particularly numerically dominant ones, have similar trends. In particular, a recent meta‐analysis of trophic control in northern hemisphere marine systems found that biodiversity, particularly when measured by higher‐order carnivores (sharks), was found to be a strong predictor of trophic control (Boyce et al., 2015).

Temperature is another factor frequently hypothesized to influence the degree of top‐down control in marine systems. Numerous studies (Boyce et al., 2015; Frank et al., 2006, 2007; Worm & Myers, 2003; Worm, Sandow, Oschlies, Lotze, & Myers, 2005) implicate temperature as a driver of large‐scale patterns in top‐down control. Specifically, Frank et al. (2006) suggest that warmer temperatures support higher demographic rates in more equatorial locations, such as intrinsic rate of increase, rendering populations inhabiting warmer waters less susceptible to overfishing. As a result, they hypothesize that these areas are less likely to show indirect effects of top‐predator loss due to fishing. Likewise, Boyce et al. (2015) found that temperature was overwhelmingly the dominant predictor of trophic control in their cross‐system meta‐analysis, explaining 32% of the total variability in trophic control. The role of temperature in governing trophic control was found to be a combination of direct and indirect effects, the latter manifesting through temperature's effect on biodiversity, omnivory, and primary productivity rate and quality (i.e., phytoplankton cell size). Differences in both temperature and seasonality between tropical and temperate areas have the potential to affect both herbivore physiology and algal growth rates that may differentially affect the propagation of top‐down effects. As with species diversity, temperature typically declines with increasing latitude, which poses an alternative or complementary explanation for latitudinal gradients in trophic control.

Fisheries harvesting from multiple trophic levels in a single ecosystem has been proposed as another explanation for the relative lack of top‐down control seen in low‐latitude systems (Newman, Paredes, Sala, & Jackson, 2006; Russ & Alcala, 1998). Many coral reef fisheries, particularly artisanal and subsistence fisheries, characteristically harvest from multiple trophic levels simultaneously (Newman et al., 2006). This style of harvest is analogous to omnivory within a food web, a factor that has long been believed to affect trophic cascade occurrence (Strong, 1992). Additionally, the more spatially constrained nature of invertebrate (e.g., urchin) versus fish herbivory could lead to greater likelihood of measurable impacts on algal assemblages in temperate areas.

Physical and biological disturbance dynamics, invasive species, and climate change in general are becoming increasingly recognized as sources of variability in community structure in tropical and temperate reef ecosystems (Brandl, Emslie, Ceccarelli, & Richards, 2016; Emslie, Cheal, & Johns, 2014; Kimbro, Byers, Grabowski, Hughes, & Piehler, 2014; Ling, Johnson, Frusher, & Ridgway, 2009; McClure et al., 2019; Mellin, Macneil, Cheal, Emslie, & Caley, 2016; Taylor et al., 2019). We cannot rule out the influence of these processes in shaping our findings, and indeed, it is almost certain that they play an increasingly important role in governing the densities of particular components of the trophic groups we examined.

As with many efforts to explain biogeographic patterns, there are many competing hypotheses that remain conceptually viable as an explanation of our results. Many of the above classes of explanations could be explored more definitively by further judicious studies across a larger geographic scales that tap the insight gained from exploring responses by different taxa, different sites, and the range of human manipulation of coastal food webs through fishing and the implementation of marine reserves. Future studies explicitly testing the mechanisms behind the patterns we describe would yield greater insight into when, and under what conditions, different types of trophic forcing will predominate. For example, examining top‐down and bottom‐up effects over latitudinal gradients in different oceans with similar oceanographic conditions but vastly different gradients in species diversity would allow the role of this hypothesized mechanism to be explicitly tested. Likewise, such replicated latitudinal gradients would allow the reproducibility of the patterns we describe to be tested, ideally in the context of both less and more heavily exploited, dynamic tropical systems (e.g., Caribbean coral reefs).

### 4.3 Alternative explanations for patterns observed

The relative lack of alternating trophic responses to significant temporal changes in predator density in the tropics (Tables S1 and S2)—regardless of fishing regulations or time‐series length—could potentially be explained by several methodological factors. In light of documented examples of top‐down control of intermediate prey species within the tropical part of our study system (e.g., Boaden & Kingsford, 2015; Graham, Evans, & Russ, 2003), we explored these methodological explanations, which include our analytical approach, our response metric (density), our time‐series durations (3–20 years, but covering up to 34‐year‐old reserves), and the subset of species surveyed at different locations.

First, the analytical approach and/or limited statistical power could prevent our metric from reliably detecting such patterns where they exist. If our approach or statistical power was inadequate, we would not necessarily expect to see any evidence of alternating population trends. However, these patterns were detected by our method in a number of locations (Table S1; Figure 5A), including the single known instance from the literature of this type of occurrence within our suite of locations (at Maria Island Reserve in temperate Tasmania, *12*).

Secondly, it is important to recognize that our metric of density, rather than biomass, renders our results conservative because a population's biomass usually responds with greater magnitude and over shorter time intervals than its number of individuals (Lester et al., 2009). Numerous other studies of top‐down effects that used density as their response metric have detected alternating trophic trends in other systems (e.g., Babcock et al., 2010; Estes & Duggins, 1995; Myers et al., 2007). Likewise, we detected such patterns using this metric in both tropical and temperate locations. Nonetheless, this is a limitation of our study and future studies would likely benefit from incorporating biomass as a response metric.

Third, time lags are known to exist between recovery of predators and measurable, top‐down effects on prey populations and, subsequently, on primary producers (Shears & Babcock, 2003; e.g., Babcock et al., 2010). Given this possibility, one might hypothesize that we detected so few cases of alternating trophic trends in the tropics because our time series (of up to 20 years) were not long enough to encompass the expected time lags. For example, Babcock et al. (2010) found that the average time to onset of indirect effects of predators was 13 years. To explicitly address this possibility, we included 15 tropical reserve locations whose surveys began 11–16 years after they were established as reserves (‘late reserves’). As a result, these locations' time series allow for up to 34 year (mean = 30.7) recovery trajectories to be examined. This combination of ‘early reserves’ and ‘late reserves’ within our tropical dataset therefore allows us to examine recovery trajectories over a collective range of 0–34 years following cessation of fishing. Our tropical locations thus span a similar range of post‐fishing‐cessation periods as our temperate locations, suggesting that the duration of our tropical locations' time series is not likely to explain differences in the prevalence of alternating trophic trends found between regions.

Lastly, our tropical surveys counted a subset of total species that did not include apex predators with low occurrence and wide home ranges (e.g., sharks; jacks [family: Carangidae]). This fact alone, however, is unlikely to explain the lack of observed alternating trophic trends given that we included all of the most numerically dominant species, including numerically dominant herbivore species' main potential predators (i.e., large snappers (family: Lutjanidae); groupers (family: Serranidae); large emperors (family: Lethrinidae)). There is no a priori expectation that significant changes in the numerically dominant predator species alone should be insufficient to lead to changes in herbivore densities or algal percent cover. Nonetheless, the different subsets of taxa included in our different study regions present a confounding factor that can be overcome with careful design of future studies.

### 4.4 Conservation and management relevance

Our findings have several key implications. First, they suggest that top‐down trophic control, in terms of numerical responses of resources to consumers, may be more apparent in our lower‐diversity, temperate marine study regions than our more speciose, tropical regions. As a result, top‐down trophic control in the form of alternating whole trophic group trends may be the exception rather than the rule (Estes et al., 2011; Ripple et al., 2014) in the tropical region of our study system. This point is supported by evidence from studies in other geographic regions within the northern hemisphere (Boyce et al., 2015; Petrie et al., 2009). Secondly, our results suggest that in the lightly to moderately exploited tropical marine system we explored, such alternating trophic trends may be a real but rare phenomenon. Future studies designed to test this finding in other tropical systems would help determine whether this pattern holds elsewhere. Lastly, our results suggest that we must carefully consider conditions under which we expect reserves to alter marine community structure and simultaneously consider local/regional context dependencies. These expectations must be clear to avoid erroneous perceptions of conservation “failures,” for example, in cases of marine reserves or other trophic rewilding (Svenning et al., 2016) activities.

## CONFLICT OF INTEREST

None declared.

## AUTHOR CONTRIBUTION

**Elizabeth M.P. Madin**: Conceptualization (lead); data curation (equal); formal analysis (equal); funding acquisition (lead); investigation (lead); methodology (lead); project administration (lead); supervision (lead); visualization (equal); writing – original draft (lead). **Joshua S. Madin**: Formal analysis (equal); methodology (supporting); visualization (equal); writing – original draft (supporting); writing – review & editing (supporting). **Aaron M.T. Harmer**: Data curation (equal); formal analysis (supporting); methodology (supporting); visualization (supporting); writing – review & editing (supporting). **Neville Barrett**: Data curation (equal); methodology (supporting); writing – review & editing (supporting). **David J. Booth**: Conceptualization (supporting); funding acquisition (supporting); supervision (supporting); writing – review & editing (supporting). **M. Julian Caley**: Data curation (equal); writing – review & editing (supporting). **Alistair J. Cheal**: Data curation (equal); methodology (supporting); writing – review & editing (supporting). **Graham J. Edgar**: Data curation (equal); methodology (supporting); writing – review & editing (supporting). **Michael J. Emslie**: Data curation (equal); methodology (supporting); writing – review & editing (supporting). **Steven D. Gaines**: Conceptualization (supporting); supervision (supporting); writing – review & editing (supporting). **Hugh P.A. Sweatman**: Data curation (equal); methodology (supporting); writing – review & editing (supporting).

## Supporting information

Figure S1

Tables S1‐S4

## Data Availability

All data and code used in this study are available for download at https://doi.org/10.5281/zenodo.3756250.
