# Supplementary material for: Latitude and protection affect decadal trends in reef trophic structure over a continental scale
Source: Ecol Evol. 2020 Jun 29;10(14):6954–66. doi: 10.1002/ece3.6347 (PMC7391320; doi:10.1002/ece3.6347)
Supplement: Supplementary file 1 — Figure S1 [file ECE3-10-6954-s001.pdf]

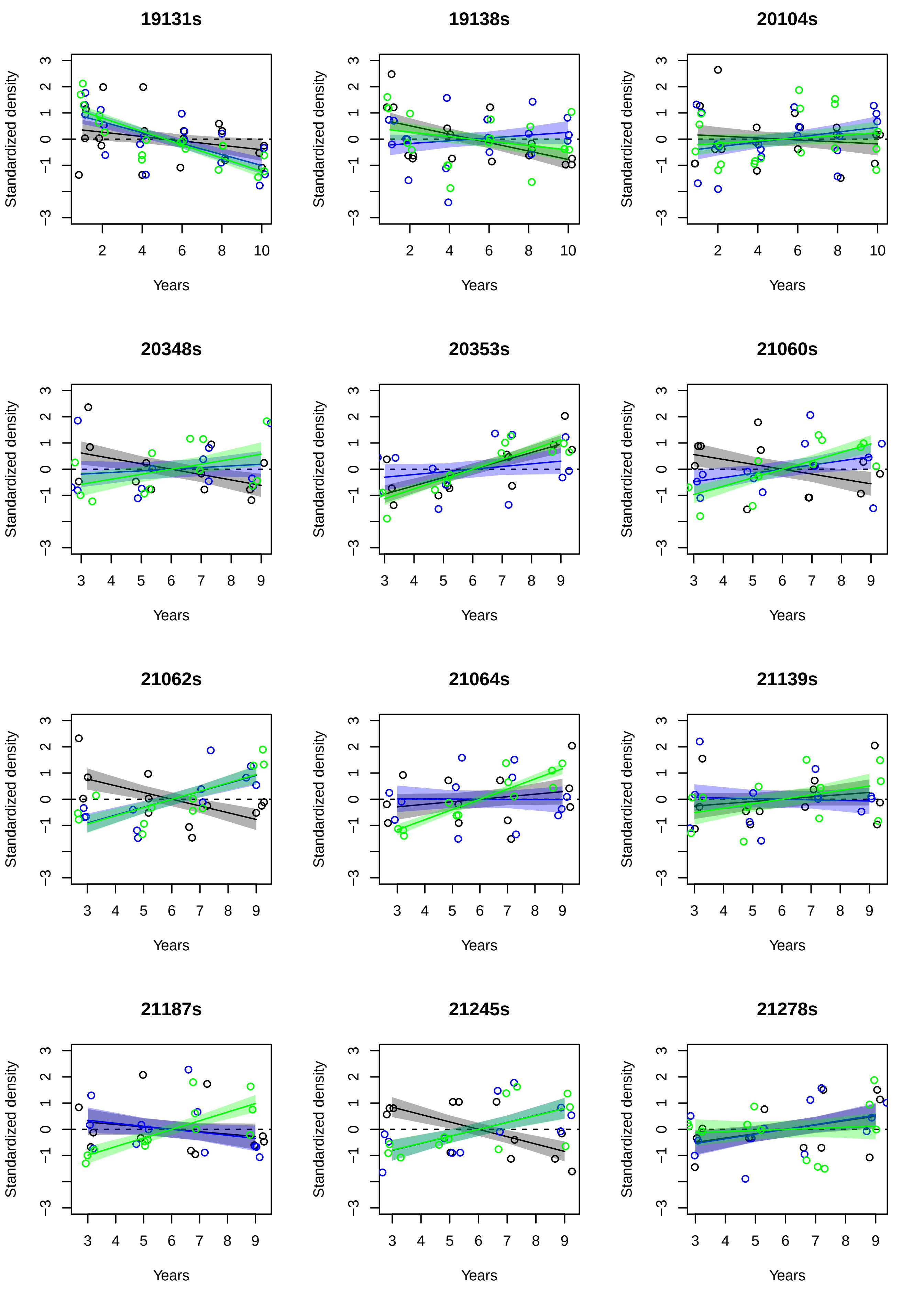
19131s
19138s
20104s
20348s
20353s
21060s
21062s
21064s
21139s
21187s
21245s
21278s
Standardized density
Years

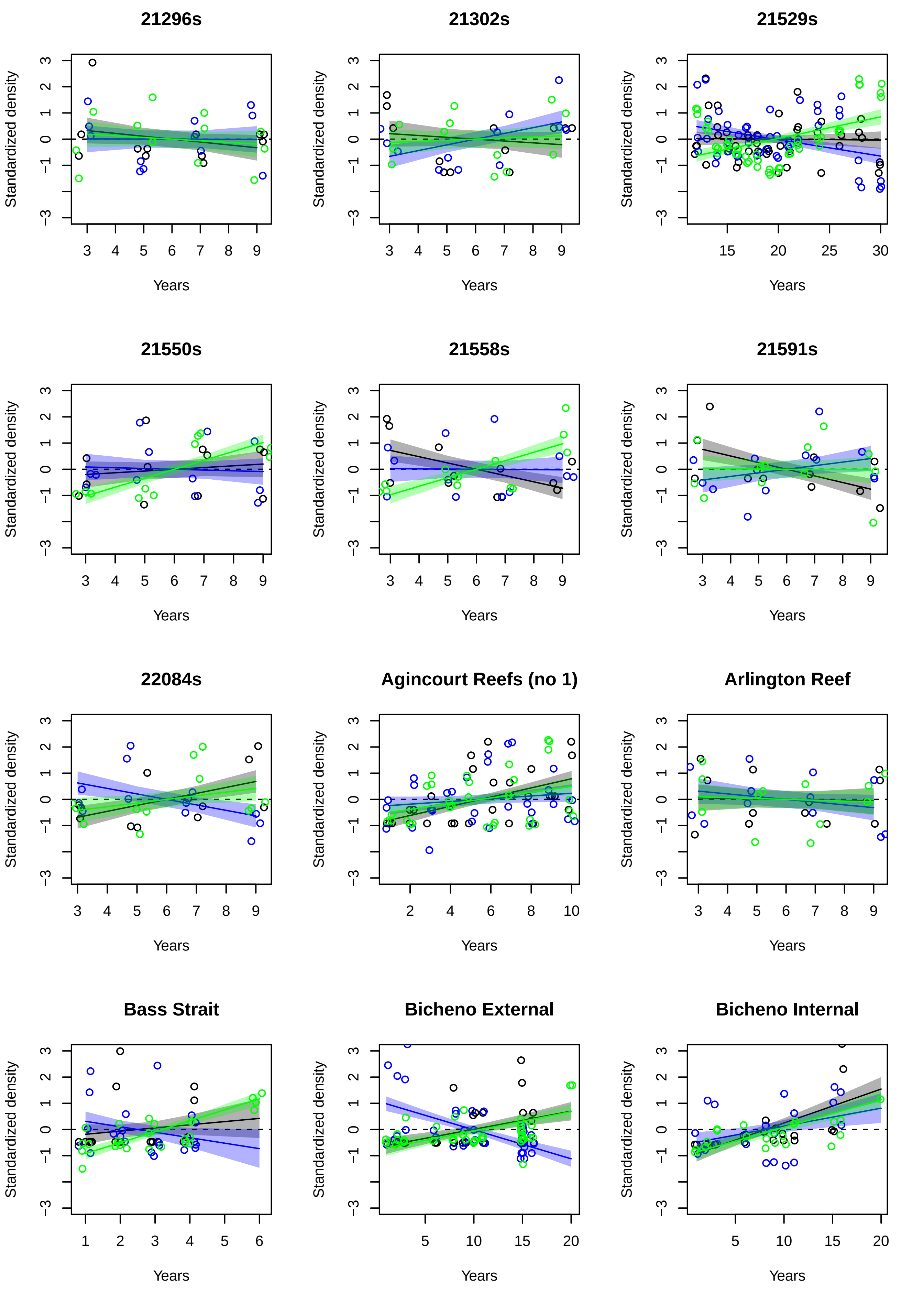
21296s
21302s
21529s
21550s
21558s
21591s
22084s
Agincourt Reefs (no 1)
Arlington Reef
Bass Strait
Bicheno External
Bicheno Internal
Standardized density
Years

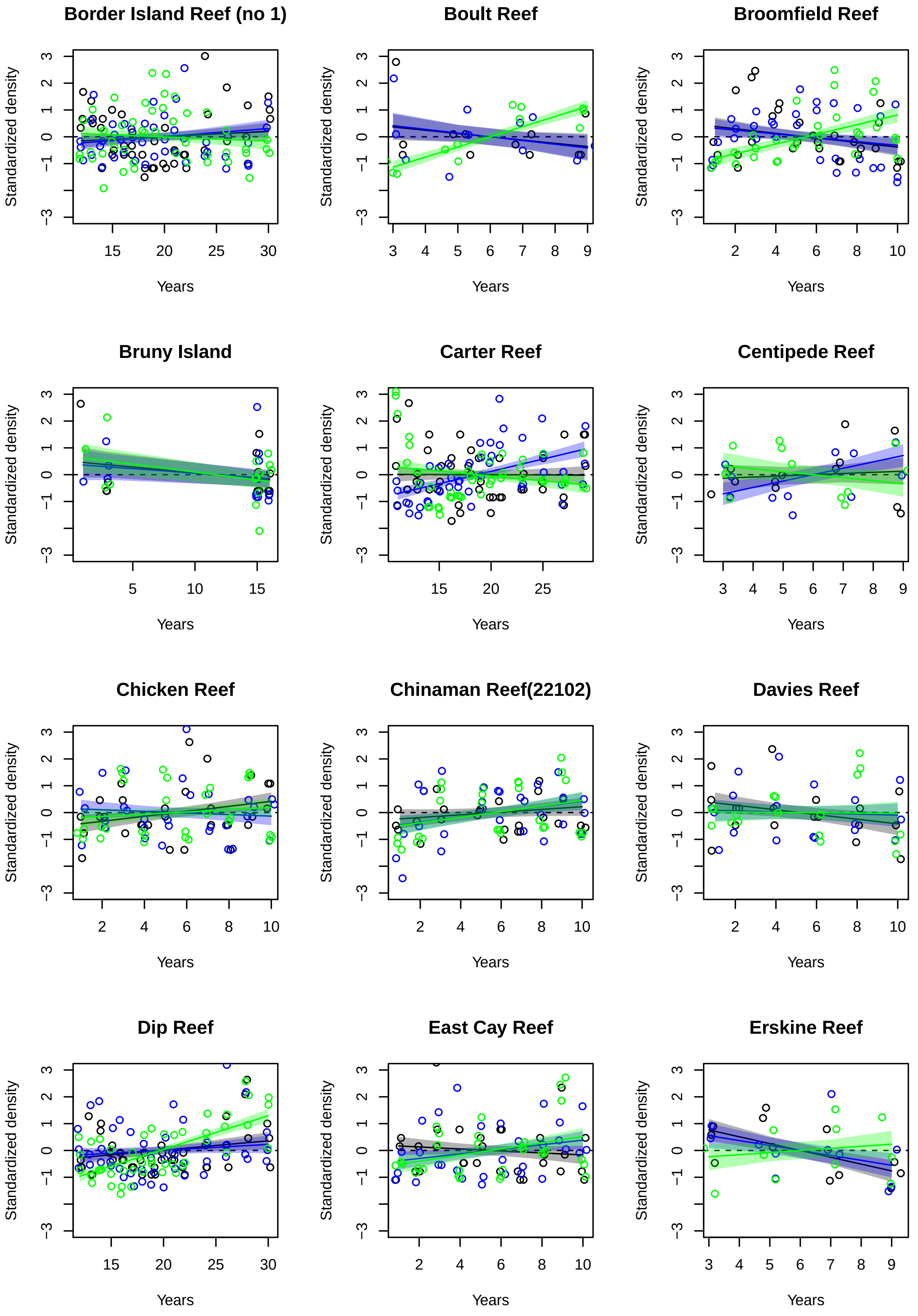
Border Island Reef (no 1)
Boult Reef
Broomfield Reef
Bruny Island
Carter Reef
Centipede Reef
Chicken Reef
Chinaman Reef(22102)
Davies Reef
Dip Reef
East Cay Reef
Erskine Reef
Standardized density
Years

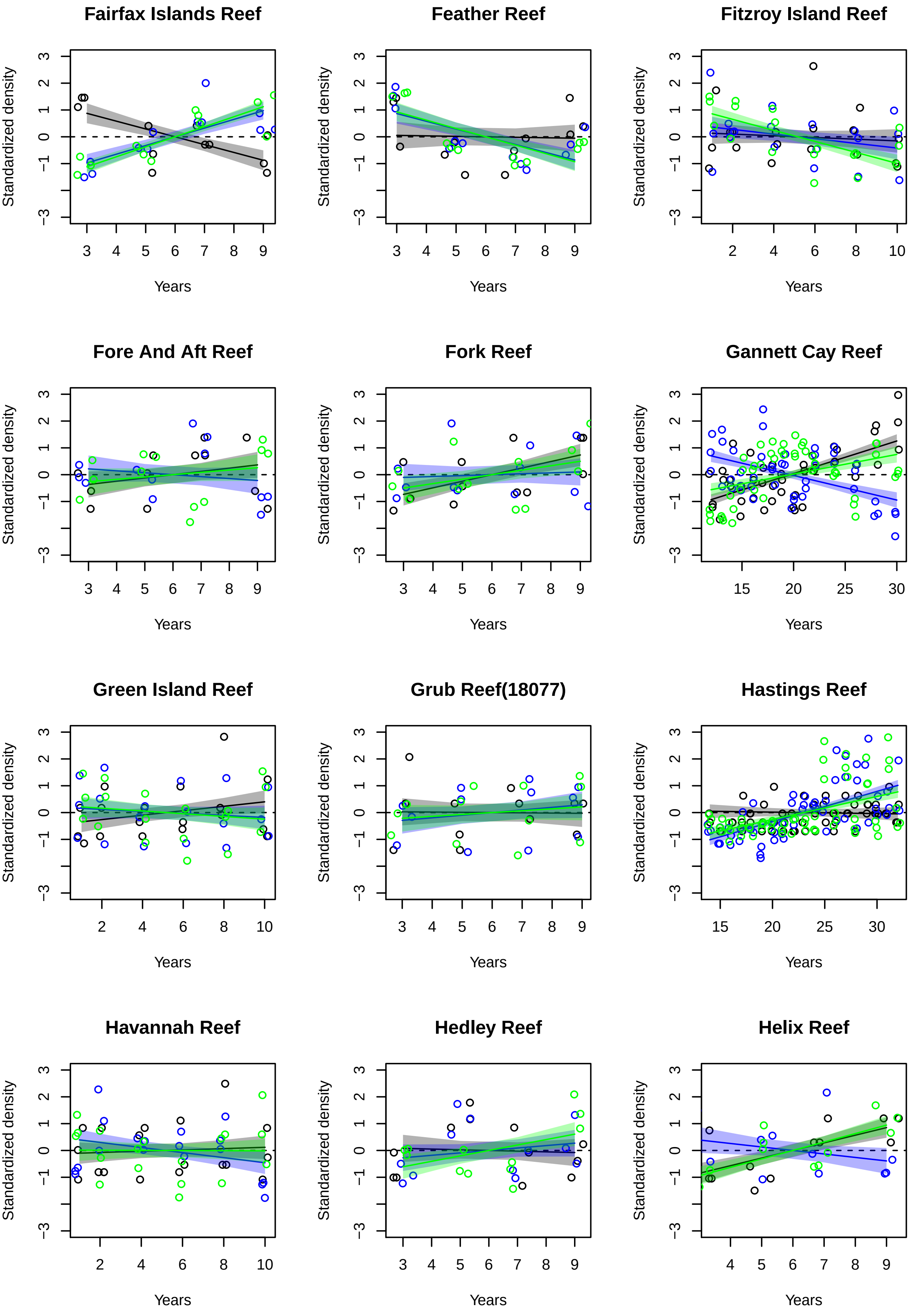

Fairfax Islands Reef
Feather Reef
Fitzroy Island Reef
Fore And Aft Reef
Fork Reef
Gannett Cay Reef
Green Island Reef
Grub Reef(18077)
Hastings Reef
Havannah Reef
Hedley Reef
Helix Reef
Standardized density
Years

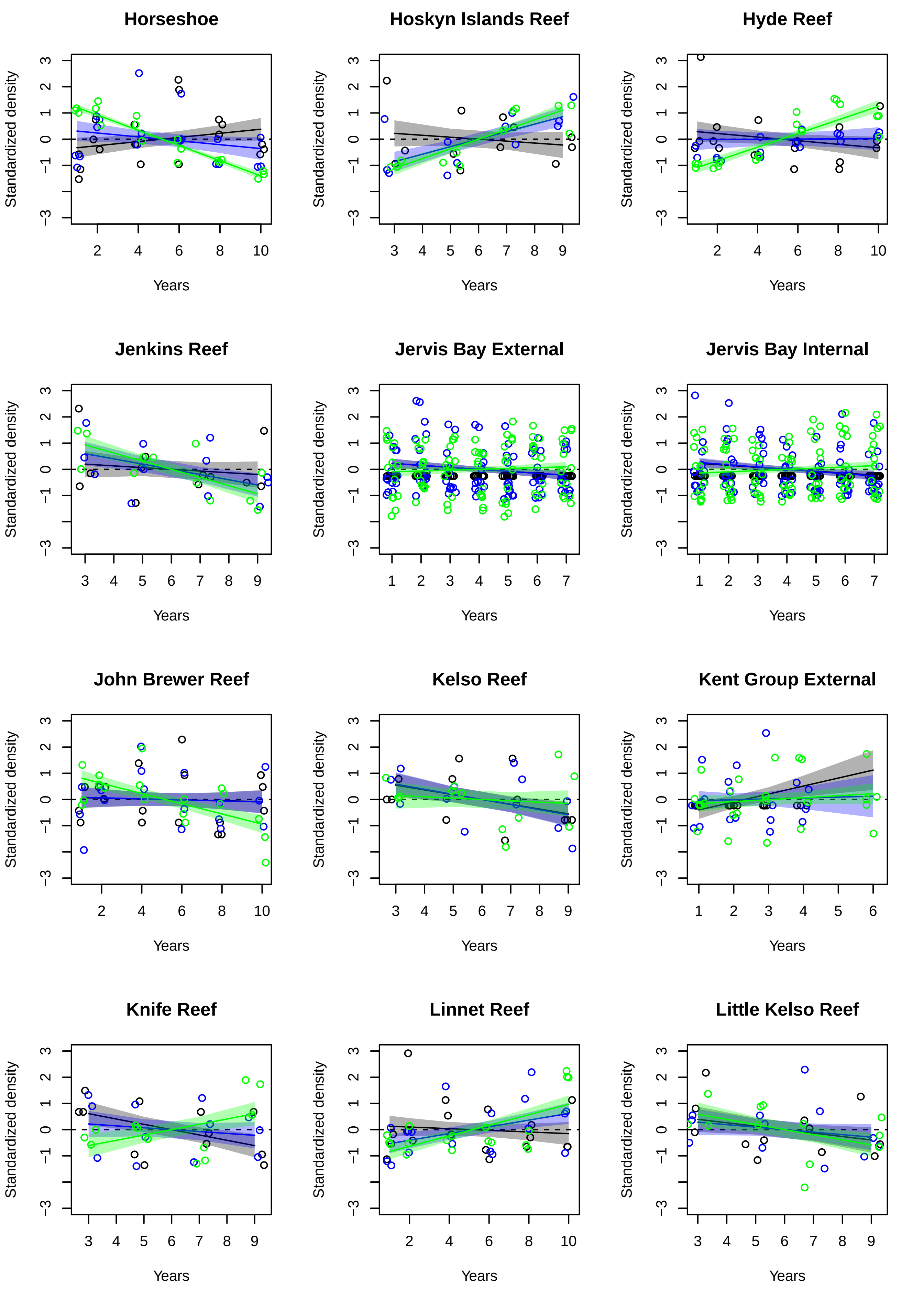

Horseshoe
Hoskyn Islands Reef
Hyde Reef
Jenkins Reef
Jervis Bay External
Jervis Bay Internal
John Brewer Reef
Kelso Reef
Kent Group External
Knife Reef
Linnet Reef
Little Kelso Reef
Standardized density
Years

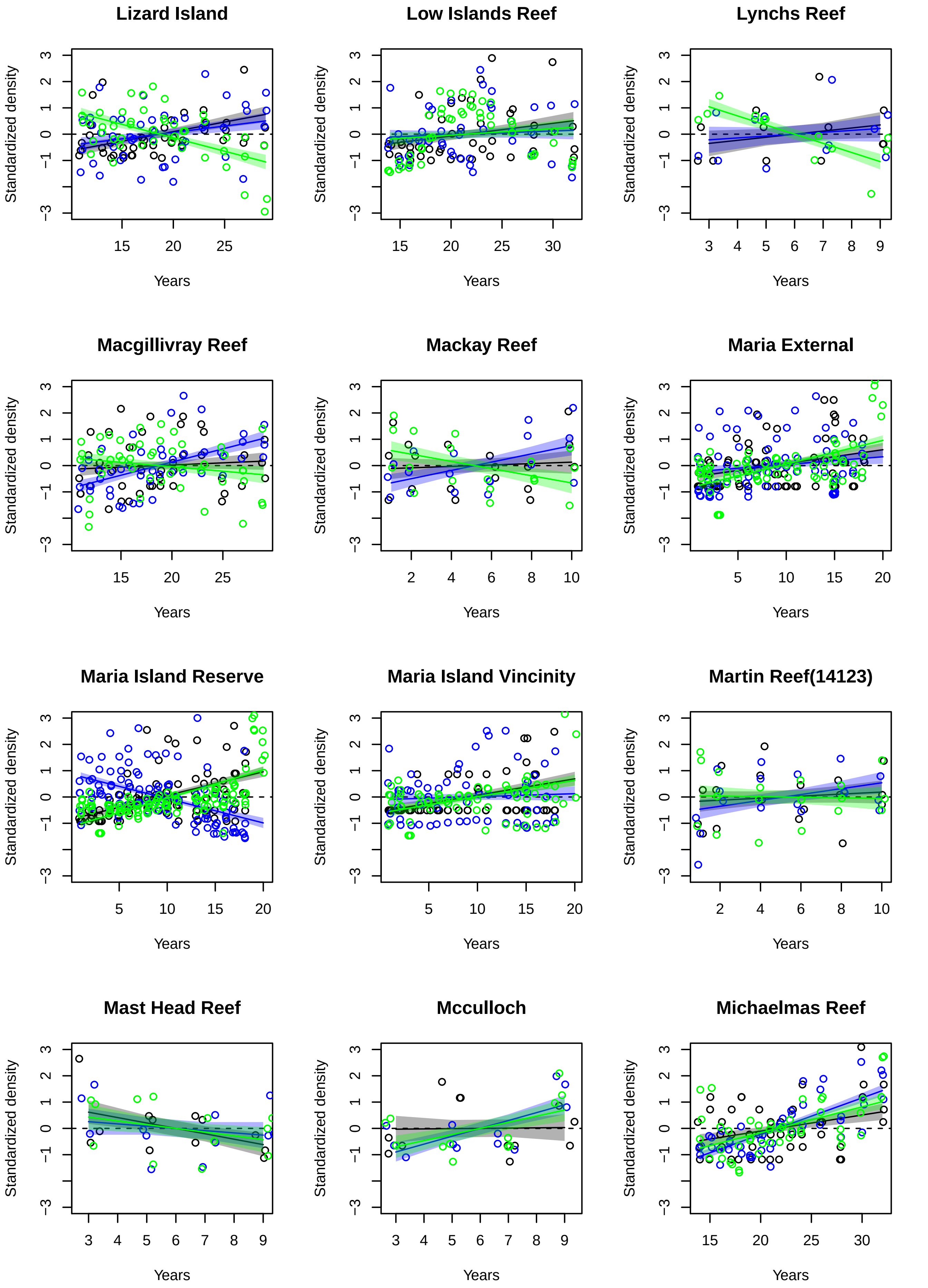
Lizard Island
Low Islands Reef
Lynchs Reef
Macgillivray Reef
Mackay Reef
Maria External
Maria Island Reserve
Maria Island Vincinity
Martin Reef(14123)
Mast Head Reef
Mcculloch
Michaelmas Reef
Standardized density
Years

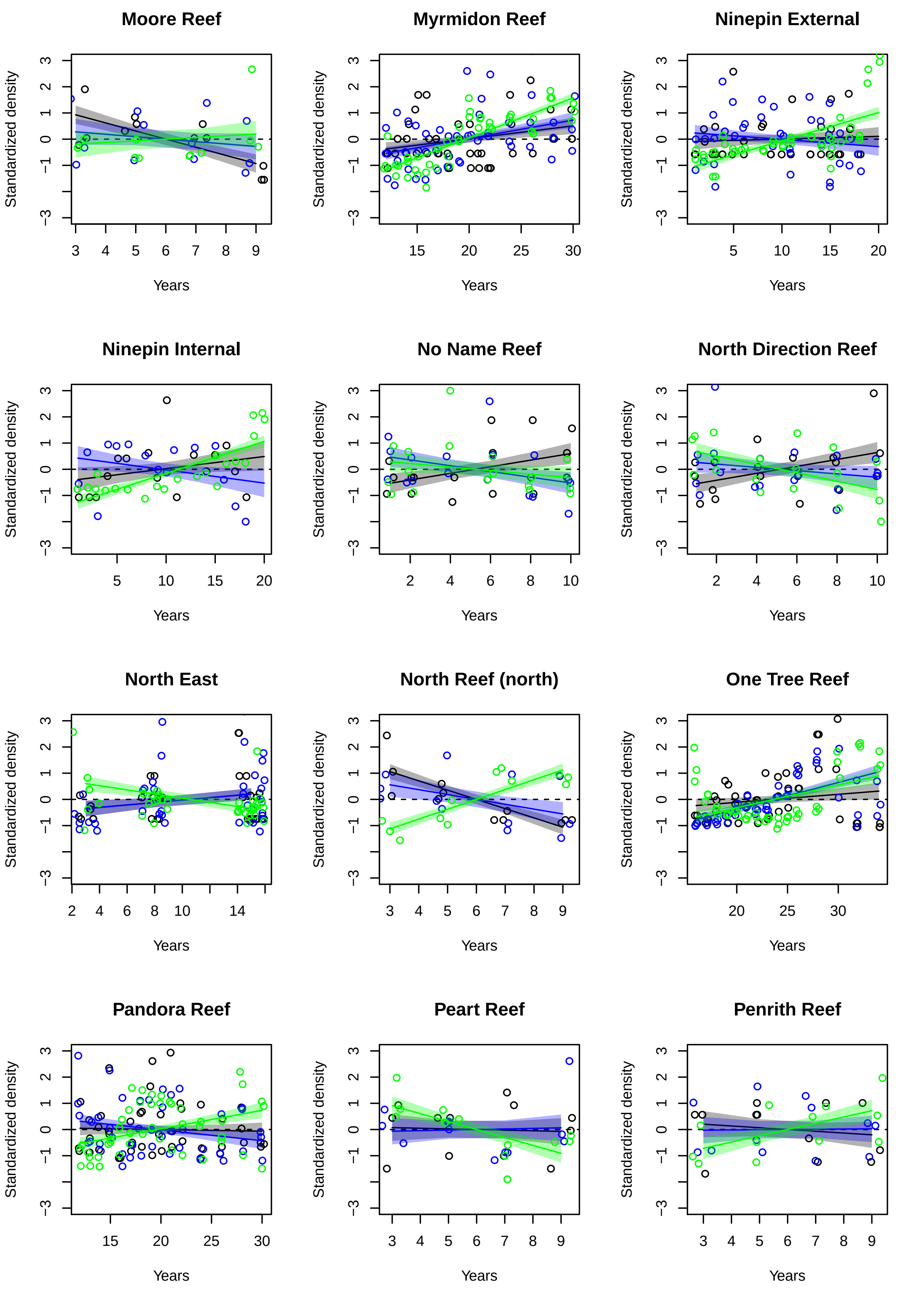
Moore Reef
Myrmidon Reef
Ninepin External
Ninepin Internal
No Name Reef
North Direction Reef
North East
North Reef (north)
One Tree Reef
Pandora Reef
Peart Reef
Penrith Reef
Standardized density
Years

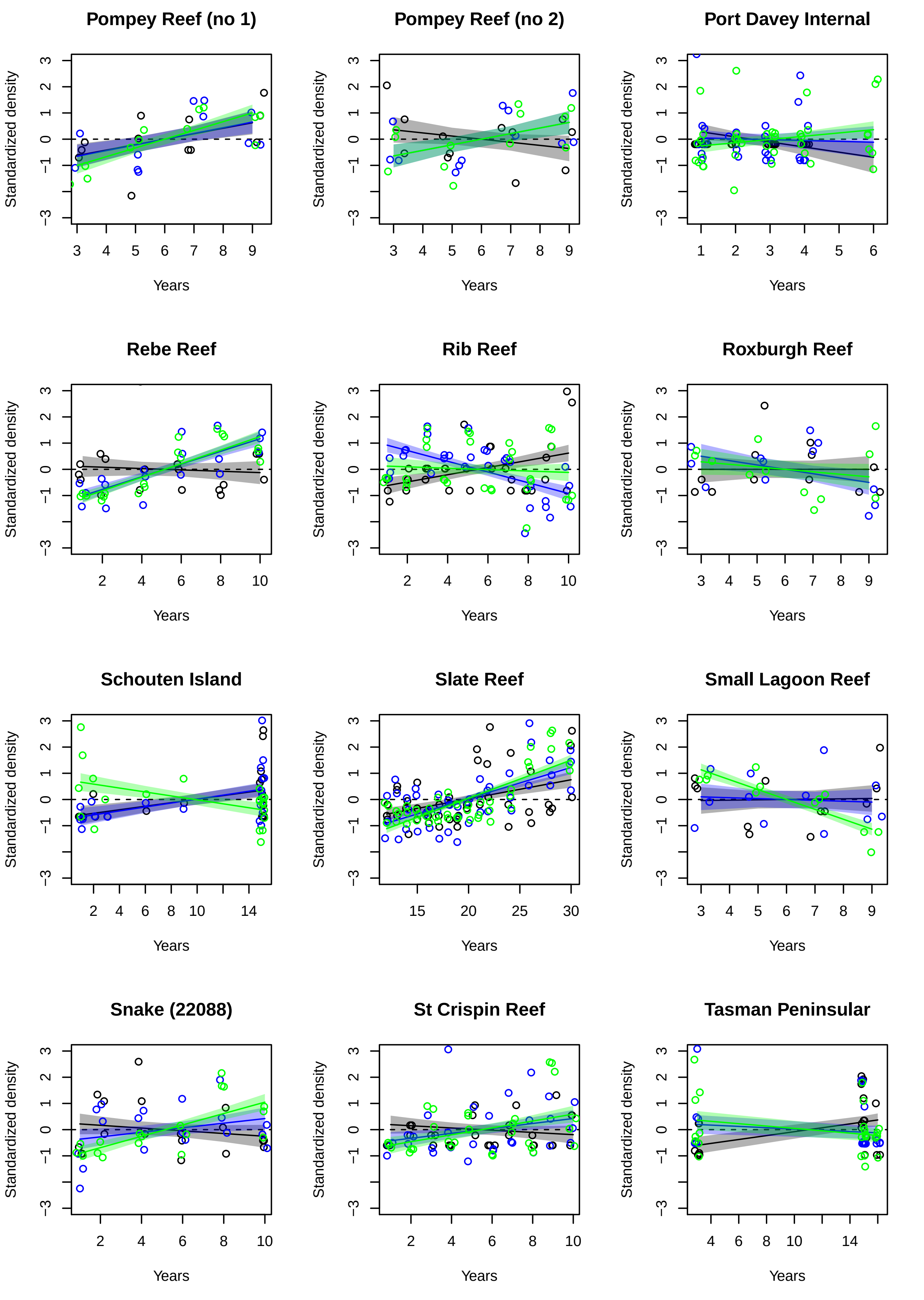

Pompey Reef (no 1)
Pompey Reef (no 2)
Port Davey Internal
Rebe Reef
Rib Reef
Roxburgh Reef
Schouten Island
Slate Reef
Small Lagoon Reef
Snake (22088)
St Crispin Reef
Tasman Peninsular
Standardized density
Years

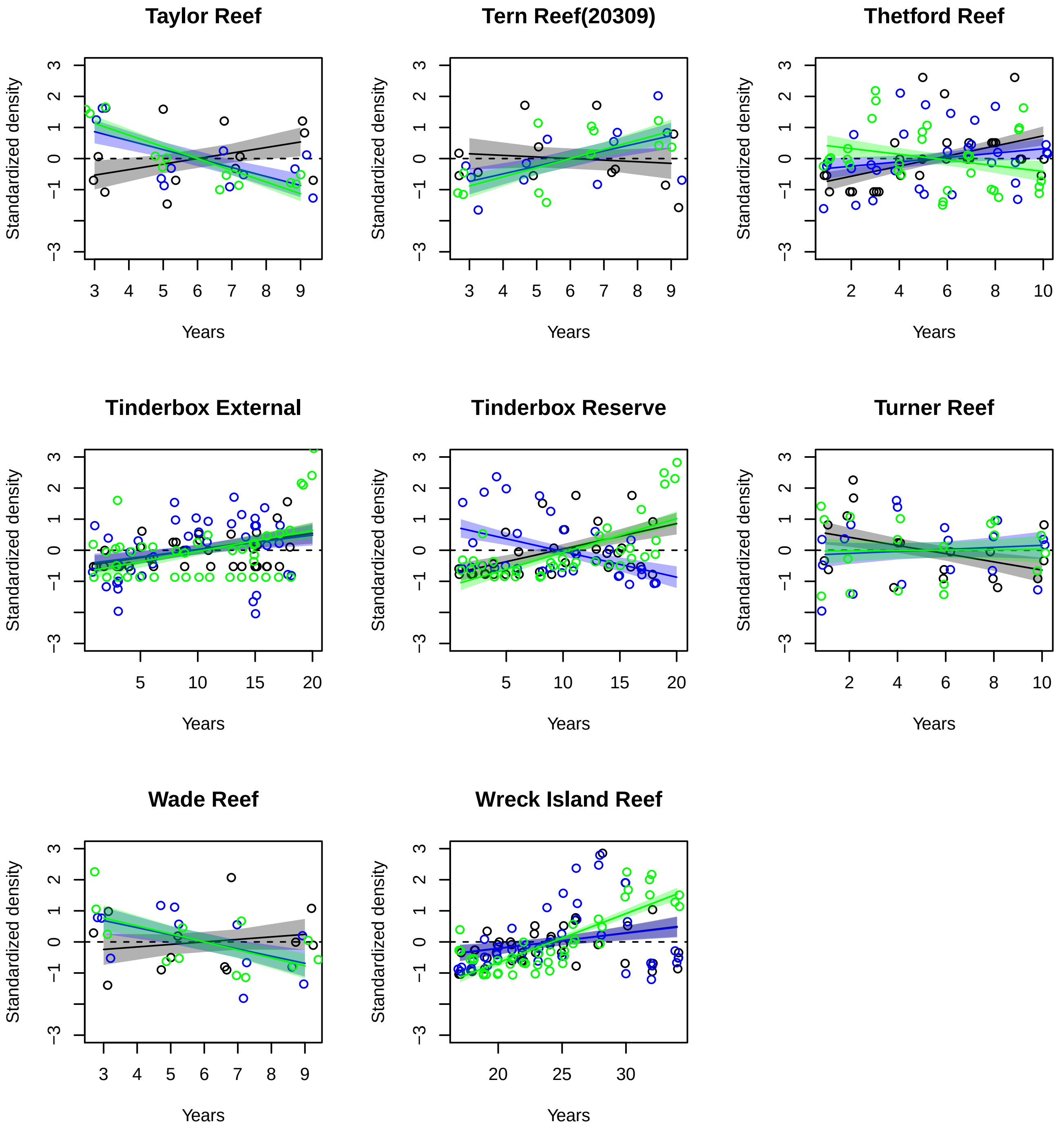

Taylor Reef
Tern Reef(20309)
Thetford Reef
Tinderbox External
Tinderbox Reserve
Turner Reef
Wade Reef
Wreck Island Reef
Standardized density
Years
