# Supplementary material for: Latitude and protection affect decadal trends in reef trophic structure over a continental scale
Source: Ecol Evol. 2020 Jun 29;10(14):6954–66. doi: 10.1002/ece3.6347 (PMC7391320; doi:10.1002/ece3.6347)
Supplement: Supplementary file 2 — Tables S1‐S4 [file ECE3-10-6954-s002.pdf]

## Supplementary tables

**Table S1.** Full summary of density trends for the three trophic groups at all 104 locations. Post-reserve survey years refer to the duration of each location's time series since reserves were established (for reserve sites) or region was rezoned (for fished sites). Climate codes: "cool_temp" is cool temperate; "warm_temp" is warm temperate. Significance codes: <0.001: "***", <0.01: "**", <0.05: "*", <0.1: ".". See *Methods* for details.

| | | | | | | | **Trend direction** | | | **Significance level** | | |
|---|---|---|---|---|---|---|---|---|---|---|---|---|
| **Location** | **Climate** | **Status** | **Part** | **State** | **Latitude** | **Post-reserve survey years** | **Predators** | **Herbivores** | **Algae** | **Predators** | **Herbivores** | **Algae** |
| Maria Island Reserve | cool_temp | protected | early | TAS | -42.57 | 20 | + | - | + | *** | *** | *** |
| Gannett Cay Reef | tropical | protected | late | QLD | -21.97 | 30 | + | - | + | *** | *** | ** |
| Tinderbox Reserve | cool_temp | protected | early | TAS | -43.05 | 20 | + | - | + | ** | ** | *** |
| Helix Reef | tropical | protected | early | QLD | -18.62 | 9 | + | - | + | * | | ** |
| Bicheno External | cool_temp | fished | always fished | TAS | -41.88 | 20 | + | - | + | * | *** | ** |
| 22084s | tropical | protected | early | QLD | -21.99 | 9 | + | - | + | . | | |
| Chicken Reef | tropical | fished | always fished | QLD | -18.65 | 10 | + | - | + | | | |
| Ninepin Internal | cool_temp | protected | early | TAS | -43.28 | 20 | + | - | + | | | *** |
| Fore And Aft Reef | tropical | fished | always fished | QLD | -18.47 | 9 | + | - | + | | | |
| 21064s | tropical | fished | always fished | QLD | -21.05 | 9 | + | - | + | | | *** |
| 21139s | tropical | protected | early | QLD | -21.45 | 9 | + | - | + | | | |
| Bass Strait | cool_temp | fished | always fished | TAS | -39.21 | 6 | + | - | + | | | *** |

| | | | | | | | | | | | | |
|---|---|---|---|---|---|---|---|---|---|---|---|---|
| 21550s | tropical | fished | always fished | QLD | -21.96 | 9 | + | - | + | | | ** |
| Ninepin External | cool_temp | fished | always fished | TAS | -43.27 | 20 | + | - | + | | | *** |
| 19138s | tropical | fished | always fished | QLD | -19.80 | 10 | - | + | - | * | | |
| 21591s | tropical | fished | always fished | QLD | -21.02 | 9 | - | + | - | * | | |
| Peart Reef | tropical | fished | always fished | QLD | -17.47 | 9 | - | + | - | | | * |
| Carter Reef | tropical | protected | late | QLD | -14.52 | 29 | - | + | - | | *** | |
| Rib Reef | tropical | fished | always fished | QLD | -18.47 | 10 | + | - | - | * | *** | |
| Tasman Peninsular | cool_temp | fished | always fished | TAS | -42.97 | 16 | + | - | - | * | | |
| North Direction Reef | tropical | protected | early | QLD | -14.74 | 10 | + | - | - | . | | * |
| No Name Reef | tropical | protected | early | QLD | -14.62 | 10 | + | - | - | . | | |
| Taylor Reef | tropical | protected | early | QLD | -17.81 | 9 | + | - | - | | * | *** |
| Green Island Reef | tropical | protected | early | QLD | -16.76 | 10 | + | - | - | | | |
| Horseshoe | tropical | fished | always fished | QLD | -22.02 | 10 | + | - | - | | | *** |
| Wade Reef | tropical | protected | early | QLD | -21.98 | 9 | + | - | - | | . | * |
| Havannah Reef | tropical | fished | always fished | QLD | -18.83 | 10 | + | - | - | | | |
| Small Lagoon Reef | tropical | fished | always fished | QLD | -21.86 | 9 | + | - | - | | | *** |
| Roxburgh Reef | tropical | fished | always fished | QLD | -18.43 | 9 | + | - | - | | | |
| Fairfax Islands Reef | tropical | protected | early | QLD | -23.84 | 9 | - | + | + | * | ** | *** |
| 21245s | tropical | fished | always fished | QLD | -21.29 | 9 | - | + | + | * | * | * |
| 21062s | tropical | fished | always fished | QLD | -21.02 | 9 | - | + | + | * | * | ** |
| Turner Reef | tropical | fished | always fished | QLD | -21.70 | 10 | - | + | + | . | | |
| 20348s | tropical | protected | early | QLD | -20.88 | 9 | - | + | + | | | |
| 21060s | tropical | fished | always fished | QLD | -21.01 | 9 | - | + | + | | | ** |
| Hyde Reef | tropical | fished | always fished | QLD | -19.74 | 10 | - | + | + | | | *** |
| Pompey Reef (no 2) | tropical | protected | early | QLD | -20.99 | 9 | - | + | + | | . | . |
| Snake (22088) | tropical | fished | always fished | QLD | -22.03 | 10 | - | + | + | | | *** |
| St Crispin Reef | tropical | fished | always fished | QLD | -16.07 | 10 | - | + | + | | | * |
| East Cay Reef | tropical | fished | always fished | QLD | -21.46 | 10 | - | + | + | | | . |
| Hoskyn Islands Reef | tropical | protected | early | QLD | -23.79 | 9 | - | + | + | | * | *** |
| 21302s | tropical | fished | always fished | QLD | -21.42 | 9 | - | + | + | | . | |
| 20104s | tropical | fished | always fished | QLD | -20.03 | 10 | - | + | + | | | |
| Penrith Reef | tropical | fished | newly fished | QLD | -21.00 | 9 | - | + | + | | | . |
| Linnet Reef | tropical | fished | always fished | QLD | -14.79 | 10 | - | + | + | | . | ** |
| Tern Reef(20309) | tropical | protected | early | QLD | -20.90 | 9 | - | + | + | | . | * |
| Rebe Reef | tropical | fished | always fished | QLD | -19.79 | 10 | - | + | + | | *** | *** |
| Hastings Reef | tropical | protected | late | QLD | -16.49 | 32 | - | + | + | | *** | *** |
| Hedley Reef | tropical | protected | early | QLD | -17.22 | 9 | - | + | + | | | |
| Grub Reef(18077) | tropical | fished | always fished | QLD | -18.62 | 9 | - | + | + | | | |
| 21529s | tropical | protected | late | QLD | -21.86 | 30 | - | - | + | | * | ** |
| Jervis Bay Internal | warm_temp | protected | early | NSW | -35.09 | 7 | - | - | + | | . | |
| Jervis Bay External | warm_temp | fished | always fished | NSW | -35.12 | 7 | - | - | + | | | |
| Pandora Reef | tropical | protected | late | QLD | -18.81 | 30 | - | - | + | | | * |

| | | | | | | | | | | | | |
|---|---|---|---|---|---|---|---|---|---|---|---|---|
| North Reef (north) | tropical | protected | early | QLD | -23.17 | 9 | - | - | + | *** | | *** |
| Erskine Reef | tropical | protected | early | QLD | -23.49 | 9 | - | - | + | * | | |
| Broomfield Reef | tropical | fished | always fished | QLD | -23.25 | 10 | - | - | + | | | ** |
| Boult Reef | tropical | fished | always fished | QLD | -23.74 | 9 | - | - | + | | | *** |
| 21187s | tropical | fished | always fished | QLD | -21.40 | 9 | - | - | + | | | ** |
| Moore Reef | tropical | protected | early | QLD | -16.84 | 9 | - | - | + | ** | | |
| Knife Reef | tropical | protected | early | QLD | -18.57 | 9 | - | - | + | | | |
| Port Davey Internal | cool_temp | protected | early | TAS | -43.33 | 6 | - | - | + | | | |
| 21558s | tropical | protected | early | QLD | -21.53 | 9 | - | - | + | . | | ** |
| Macgillivray Reef | tropical | protected | late | QLD | -14.65 | 29 | + | + | - | | *** | |
| Schouten Island | cool_temp | fished | always fished | TAS | -42.31 | 15 | + | + | - | . | * | * |
| Mackay Reef | tropical | fished | always fished | QLD | -16.04 | 10 | + | + | - | | * | . |
| Centipede Reef | tropical | fished | always fished | QLD | -18.73 | 9 | + | + | - | | . | |
| Lizard Island | tropical | protected | late | QLD | -14.69 | 29 | + | + | - | ** | . | *** |
| Martin Reef(14123) | tropical | fished | always fished | QLD | -14.75 | 10 | + | + | - | | | |
| North East | cool_temp | fished | always fished | TAS | -41.34 | 15 | + | + | - | | | * |
| Thetford Reef | tropical | fished | always fished | QLD | -16.79 | 10 | + | + | - | ** | | |
| Border Island Reef (no 1) | tropical | protected | late | QLD | -20.18 | 30 | + | + | - | | | |
| Lynchs Reef | tropical | protected | early | QLD | -18.73 | 9 | + | + | - | | | ** |
| Bicheno Internal | cool_temp | protected | early | TAS | -41.87 | 20 | + | + | + | ** | | ** |
| Agincourt Reefs (no 1) | tropical | protected | early | QLD | -16.04 | 10 | + | + | + | ** | | . |
| Maria Island Vincinity | cool_temp | fished | always fished | TAS | -42.50 | 20 | + | + | + | ** | | ** |
| 20353s | tropical | protected | early | QLD | -20.95 | 9 | + | + | + | ** | | *** |
| Slate Reef | tropical | protected | late | QLD | -19.66 | 30 | + | + | + | ** | *** | *** |
| Maria External | cool_temp | fished | always fished | TAS | -42.57 | 20 | + | + | + | * | | *** |
| Michaelmas Reef | tropical | protected | late | QLD | -16.55 | 32 | + | + | + | * | *** | *** |
| Fork Reef | tropical | protected | early | QLD | -18.60 | 9 | + | + | + | . | | |
| Tinderbox External | cool_temp | fished | always fished | TAS | -43.03 | 20 | + | + | + | . | | ** |
| Myrmidon Reef | tropical | protected | late | QLD | -18.25 | 30 | + | + | + | . | * | *** |
| Low Islands Reef | tropical | protected | late | QLD | -16.39 | 32 | + | + | + | . | | |
| Wreck Island Reef | tropical | protected | late | QLD | -23.31 | 34 | + | + | + | . | | *** |
| Pompey Reef (no 1) | tropical | protected | early | QLD | -20.92 | 9 | + | + | + | | . | ** |
| Kent Group External | cool_temp | fished | always fished | TAS | -39.45 | 6 | + | + | + | | | |
| 21278s | tropical | protected | early | QLD | -21.10 | 9 | + | + | + | | | |
| Dip Reef | tropical | protected | late | QLD | -18.40 | 30 | + | + | + | | | *** |
| One Tree Reef | tropical | protected | late | QLD | -23.48 | 34 | + | + | + | | *** | ** |
| Chinaman Reef(22102) | tropical | fished | always fished | QLD | -21.99 | 10 | + | + | + | | | |
| Mcculloch | tropical | fished | always fished | QLD | -17.28 | 9 | + | + | + | | * | . |
| Mast Head Reef | tropical | fished | always fished | QLD | -23.53 | 9 | - | - | - | | | |
| Kelso Reef | tropical | protected | early | QLD | -18.42 | 9 | - | - | - | | | |
| Davies Reef | tropical | fished | newly fished | QLD | -18.80 | 10 | - | - | - | | | |
| 19131s | tropical | fished | always fished | QLD | -19.76 | 10 | - | - | - | | ** | *** |

| | | | | | | | | | | | | |
|---|---|---|---|---|---|---|---|---|---|---|---|---|
| Little Kelso Reef | tropical | protected | early | QLD | -18.46 | 9 | - | - | - | | | |
| Bruny Island | cool temp | fished | always fished | TAS | -43.18 | 16 | - | - | - | | | |
| 21296s | tropical | protected | early | QLD | -21.32 | 9 | - | - | - | | | |
| Jenkins Reef | tropical | protected | early | QLD | -21.95 | 9 | - | - | - | | | ** |
| Fitzroy Island Reef | tropical | fished | always fished | QLD | -16.92 | 10 | - | - | - | | | ** |
| John Brewer Reef | tropical | fished | newly fished | QLD | -18.62 | 10 | - | - | - | | | ** |
| Arlington Reef | tropical | fished | always fished | QLD | -16.64 | 9 | - | - | - | | | |
| Feather Reef | tropical | protected | early | QLD | -17.51 | 9 | - | - | - | | * | ** |

**Table S2.** Model selection results following Zuur *et al.* (8) using the *drop1* function in R on the 24 locations with significant changes in predator densities through time (Table S1).. LRT refers to likelihood ratio test.

| **Cascades** | *Deviance* | *AIC* | *LRT* | *p-value* | |
|---|---|---|---|---|---|
| (Null) | 9.48 | 17.48 | NA | NA | |
| Latitude | 21.01 | 27.01 | 11.53 | 0.001 | * |
| Exploitation status | 16.72 | 20.72 | 7.24 | 0.027 | * |
| **Predators** | *SS* | *AIC* | *F value* | *p-value* | |
| (Null) | NA | 91.11 | NA | NA | |
| Latitude | 22.97 | 99.14 | 10.02 | 0.002 | * |
| Exploitation status | 23.95 | 95.55 | 3.48 | 0.019 | * |
| **Herbivores** | | | | | |
| (Null) | NA | 125.58 | NA | NA | |
| Latitude | 14.37 | 128.2 | 4.5 | 0.036 | * |
| Exploitation status | 39.59 | 131.85 | 4.13 | 0.008 | * |
| **Algae** | | | | | |
| (Null) | NA | 206.26 | NA | NA | |

| | | | | | |
|---|---|---|---|---|---|
| Latitude | 49.56 | 211.51 | 7.15 | 0.009 | * |
| Exploitation status | 48.38 | 207.34 | 2.33 | 0.079 | |

**Table S3.** Model results for generalized linear model (GLM; for alternating trophic trend pattern prevalence) and linear mixed effect models (LMMs; for individual trophic group trends). GLM uses a binomial response variable (0: no alternating trophic trends associated with significant predator trend; 1: alternating trophic trends associated with significant predator trend) and the logit link function. Two separate models, one for latitude and one for exploitation status, were run for each of alternating trophic trend prevalence and individual trophic groups. LMMs include location as a random effect. Significance codes: <0.001: "***", <0.01: "**", <0.05: "*", <0.1: ".". *Pr(>|z|)* denotes p-value.

| **Cascades** | *Estimate* | *Std. Error* | *z value* | *Pr(>\|z\|)* |
|---|---|---|---|---|
| (Intercept) | -5.17 | 2.15 | -2.4 | 0.016* |
| Latitude | -0.12 | 0.06 | -1.97 | 0.048* |
| (Always fished) | -2.2 | 1.05 | -2.08 | 0.037* |
| Early | 0.81 | 1.32 | 0.62 | 0.538 |
| Late | 1.10 | 1.56 | 0.70 | 0.482 |
| **Predators** | | | | |
| (Intercept) | -0.05 | 0.09 | -0.55 | 0.583 |
| Latitude | -0.01 | 0 | -2.08 | 0.040* |
| (Always fished) | 0.07 | 0.05 | 1.32 | 0.191 |
| Early | 0.07 | 0.08 | 0.9 | 0.369 |
| Late | 0.14 | 0.08 | 1.8 | 0.075 |
| Newly fished | -0.24 | 0.26 | -0.92 | 0.361 |
| **Herbivores** | | | | |
| (Intercept) | 0.4 | 0.1 | 3.87 | 0.000 * |

| | | | | |
|---|---|---|---|---|
| Latitude | 0.01 | 0 | 3.61 | 0.000 * |
| (Always fished) | 0.04 | 0.06 | 0.73 | 0.466 |
| Early | -0.21 | 0.09 | -2.32 | 0.023 * |
| Late | 0.26 | 0.09 | 2.80 | 0.006 * |
| Newly fished | -0.09 | 0.03 | -0.29 | 0.770 |
| **Algae** | | | | |
| (Intercept) | 0.03 | 0.15 | 0.22 | 0.824 |
| Latitude | -0.01 | 0.01 | -1.9 | 0.060 |
| (Always fished) | 0.21 | 0.08 | 2.49 | 0.014 * |
| Early | 0.14 | 0.14 | 1.03 | 0.304 |
| Late | 0.19 | 0.14 | 1.42 | 0.160 |
| Newly fished | -0.37 | 0.44 | -0.83 | 0.411 |

**Table S4.** List of species included in each trophic/functional group. "Predators" represent piscivorous fishes in topical locations and large benthic invertivorous fishes (>30 cm TL) and invertebrates (lobsters > 10 cm CP) in temperate locations. This list includes all species counted in surveys, but only those that met the minimum size thresholds were included in analyses. "Herbivores" represent herbivorous fishes in tropical locations and herbivorous invertebrates in temperate locations. "Algae" represent turf and fleshy macroalgae in tropical locations and canopy-forming brown algae and foliose brown, green, and red algae in temperate locations. For details of criteria underlying species inclusion, see *Methods*.

| **Species** | **Functional group** |
|---|---|
| *Acrocarpia paniculata* | algae |
| *Acrocarpia robusta* | algae |
| *Acrosorium ciliolatum* | algae |
| *Amphiroa anceps* | algae |
| *Amphiroa gracilis* | algae |
| *Amphiroa* spp. | algae |
| *Apjohnia laetevirens* | algae |
| *Apoglossum spathulatum* | algae |
| *Areschougia congesta* | algae |
| *Areschougia* spp. | algae |
| *Arthrocardia wardii* | algae |
| *Asperococcus bullosus* | algae |
| *Bellotia eriophorum* | algae |
| *Callophycus laxus* | algae |
| *Callophycus* spp. | algae |
| *Carpoglossum confluens* | algae |
| *Carpomitra costata* | algae |
| *Carpopeltis elata* | algae |

| | |
|---|---|
| *Carpopeltis phyllophora* | algae |
| *Caulerpa annulata* | algae |
| *Caulerpa brownii* | algae |
| *Caulerpa cactoides* | algae |
| *Caulerpa flexilis* | algae |
| *Caulerpa flexilis* var. *muelleri* | algae |
| *Caulerpa geminata* | algae |
| *Caulerpa hodgkinsoniae* | algae |
| *Caulerpa longifolia* | algae |
| *Caulerpa obscura* | algae |
| *Caulerpa remotifolia* | algae |
| *Caulerpa scalpelliformis* | algae |
| *Caulerpa simpliciuscula* | algae |
| *Caulerpa* spp. | algae |
| *Caulerpa trifaria* | algae |
| *Caulerpa vesiculifera* | algae |
| *Caulocystis cephalornithos* | algae |
| *Caulocystis* spp. | algae |
| *Caulocystis uvifera* | algae |
| *Chlanidophora microphylla* | algae |

| | |
|---|---|
| *Cladostephus spongiosus* | algae |
| *Claviclonium ovatum* | algae |
| *Codium australicum* | algae |
| *Codium cuneatum* | algae |
| *Codium dimorphum* | algae |
| *Codium duthieae* | algae |
| *Codium fragile* | algae |
| *Codium fragile* subsp. *tomentosoides* | algae |
| *Codium galeatum* | algae |
| *Codium harveyi* | algae |
| *Codium lucasii* | algae |
| *Codium perriniae* | algae |
| *Codium pomoides* | algae |
| *Codium spongiosum* | algae |
| *Codium* spp. | algae |
| *Colpomenia peregrina* | algae |
| *Colpomenia sinuosa* | algae |
| *Colpomenia* spp. | algae |
| *Curdiea angustata* | algae |
| *Curdiea obesa* | algae |

| | |
|---|---|
| *Cystophora brownii* | algae |
| *Cystophora congesta* | algae |
| *Cystophora expansa* | algae |
| *Cystophora grevillei* | algae |
| *Cystophora harveyi* | algae |
| *Cystophora intermedia* | algae |
| *Cystophora monilifera* | algae |
| *Cystophora moniliformis* | algae |
| *Cystophora pectinata* | algae |
| *Cystophora platylobium* | algae |
| *Cystophora polycystidea* | algae |
| *Cystophora racemosa* | algae |
| *Cystophora retorta* | algae |
| *Cystophora retroflexa* | algae |
| *Cystophora siliquosa* | algae |
| *Cystophora* spp. | algae |
| *Cystophora subfarcinata* | algae |
| *Cystophora xiphocarpa* | algae |
| *Delisea elegans* | algae |
| *Delisea hypneoides* | algae |

| | |
|---|---|
| *Delisea plumosa* | algae |
| *Delisea pulchra* | algae |
| *Delisea* spp. | algae |
| *Desmarestia ligulata* | algae |
| *Dictyomenia angusta* | algae |
| *Dictyomenia harveyana* | algae |
| *Dictyomenia sonderi* | algae |
| *Dictyomenia tridens* | algae |
| *Dictyopteris acrostichoides* | algae |
| *Dictyopteris australis* | algae |
| *Dictyopteris muelleri* | algae |
| *Dictyopteris* spp. | algae |
| *Dictyota dichotoma* | algae |
| *Dictyota diemensis* | algae |
| *Dictyota* spp. | algae |
| *Dictyotaceae* spp. | algae |
| *Dilophus gunnianus* | algae |
| *Dilophus marginatus* | algae |
| *Dilophus* spp. | algae |
| *Distromium flabellatum* | algae |

| | |
|---|---|
| *Distromium multifidum* | algae |
| *Distromium* spp. | algae |
| *Durvillaea potatorum* | algae |
| *Ecklonia radiata* | algae |
| *Erythrymenia minuta* | algae |
| *Galaxaura marginata* | algae |
| *Gelinaria ulvoidea* | algae |
| *Gigartina brachiata* | algae |
| *Gigartina crassicaulis* | algae |
| *Gigartina muelleriana* | algae |
| *Gigartina sonderi* | algae |
| *Gigartina* spp. | algae |
| *Gloiosaccion brownii* | algae |
| *Glossophora nigricans* | algae |
| *Grateloupia filicina* | algae |
| *Grateloupia turuturu* | algae |
| *Halicnide similans* | algae |
| *Haloplegma preissii* | algae |
| *Halopteris paniculata* | algae |
| *Halopteris* spp. | algae |

| | |
|---|---|
| *Halymenia plana* | algae |
| *Halymenia* spp. | algae |
| *Hemineura frondosa* | algae |
| *Heterodoxia denticulata* | algae |
| *Heterodoxia* spp. | algae |
| *Homoeostrichus olsenii* | algae |
| *Homoeostrichus sinclairii* | algae |
| *Hormosira banksii* | algae |
| *Hymenena affinis* | algae |
| *Hymenena curdieana* | algae |
| *Hymenena* spp. | algae |
| *Jeannerettia lobata* | algae |
| *Jeannerettia pedicellata* | algae |
| *Kallymenia cribrosa* | algae |
| *Kallymenia* spp. | algae |
| *Kallymenia tasmanica* | algae |
| *Laurencia botryoides* | algae |
| *Laurencia brongniartii* | algae |
| *Laurencia clavata* | algae |
| *Laurencia elata* | algae |

| | |
|---|---|
| *Laurencia filiformis* | algae |
| *Laurencia majuscula* | algae |
| *Laurencia* spp. | algae |
| *Laurencia tasmanica* | algae |
| *Leathesia difformis* | algae |
| *Lenormandia marginata* | algae |
| *Lenormandia muelleri* | algae |
| *Lenormandia spectabilis* | algae |
| *Lenormandia* spp. | algae |
| *Lessonia corrugata* | algae |
| *Lobophora variegata* | algae |
| *Lobospira bicuspidata* | algae |
| *Macrocystis angustifolia* | algae |
| *Macrocystis pyrifera* | algae |
| *Martensia australis* | algae |
| *Martensia fragilis* | algae |
| *Melanthalia abscissa* | algae |
| *Melanthalia concinna* | algae |
| *Melanthalia obtusata* | algae |
| *Melanthalia* spp. | algae |

| | |
|---|---|
| *Metamastophora flabellata* | algae |
| *Myriodesma integrifolium* | algae |
| *Myriogramme gunniana* | algae |
| *Nemastoma feredayae* | algae |
| *Nitospinosa pristoidea* | algae |
| *Nitospinosa tasmanica* | algae |
| *Padina fraseri* | algae |
| *Padina* spp. | algae |
| *Perithalia caudata* | algae |
| *Phacelocarpus alatus* | algae |
| *Phacelocarpus apodus* | algae |
| *Phacelocarpus peperocarpos* | algae |
| *Phacelocarpus sessilis* | algae |
| *Phacelocarpus* spp. | algae |
| *Phyllospora comosa* | algae |
| *Platythalia angustifolia* | algae |
| *Plocamium angustum* | algae |
| *Plocamium cartilagineum* | algae |
| *Plocamium costatum* | algae |
| *Plocamium dilatatum* | algae |

| | |
|---|---|
| *Plocamium leptophyllum* | algae |
| *Plocamium mertensii* | algae |
| *Plocamium patagiatum* | algae |
| *Plocamium preissianum* | algae |
| *Pollexfenia* spp. | algae |
| *Polycoelia laciniata* | algae |
| *Pterocladia lucida* | algae |
| *Ptilonia australasica* | algae |
| *Ptilonia subulifera* | algae |
| *Ptilota hannafordii* | algae |
| *Rhodoglossum gigartinoides* | algae |
| *Rhodoglossum* spp. | algae |
| *Rhodopeltis australis* | algae |
| *Rhodophyllis membranacea* | algae |
| *Rhodymenia cuneata* | algae |
| *Rhodymenia leptophylla* | algae |
| *Rhodymenia obtusa* | algae |
| *Rhodymenia prolificans* | algae |
| *Rhodymenia sonderi* | algae |
| *Rhodymenia* spp. | algae |

| | |
|---|---|
| *Rhodymenia stenoglossa* | algae |
| *Rhodymenia verrucosa* | algae |
| *Sargassum decipiens* | algae |
| *Sargassum distichum* | algae |
| *Sargassum fallax* | algae |
| *Sargassum heteromorphum* | algae |
| *Sargassum lacerifolium* | algae |
| *Sargassum linearifolium* | algae |
| *Sargassum paradoxum* | algae |
| *Sargassum sonderi* | algae |
| *Sargassum spinuligerum* | algae |
| *Sargassum* spp. | algae |
| *Sargassum varians* | algae |
| *Sargassum verruculosum* | algae |
| *Sargassum vestitum* | algae |
| *Scaberia agardhii* | algae |
| *Schizymenia* spp. | algae |
| *Seirococcus axillaris* | algae |
| *Solieria robusta* | algae |
| *Sonderopelta coriacea* | algae |

| | |
|---|---|
| *Sonderopelta/Peyssonnelia* | algae |
| *Sporochnus comosus* | algae |
| *Sporochnus* spp. | algae |
| *Stenogramme interrupta* | algae |
| *Stypopodium flabelliforme* | algae |
| *Taonia australasica* | algae |
| *Thamnoclonium dichotomum* | algae |
| *Thamnophyllis lacerata* | algae |
| *Thuretia quercifolia* | algae |
| *Tsengia feredayae* | algae |
| *Tylotus obtusatus* | algae |
| *Ulva rigida* | algae |
| *Ulva* spp. | algae |
| *Undaria pinnatifida* | algae |
| *Xiphophora chondrophylla* | algae |
| *Xiphophora gladiata* | algae |
| *Zonaria angustata* | algae |
| *Zonaria diesingiana* | algae |
| *Zonaria spiralis* | algae |
| *Zonaria* spp. | algae |

| | |
|---|---|
| *Zonaria turneriana/angustata* | algae |
| *Asymbolus analis* | predator |
| *Cheilodactylus nigripes* | predator |
| *Cheilodactylus spectabilis* | predator |
| *Cnidoglanis macrocephalus* | predator |
| *Dactylophora nigricans* | predator |
| *Dasyatis brevicaudata* | predator |
| *Dasyatis thetidis* | predator |
| *Gymnothorax prasinus* | predator |
| *Heterodontus portusjacksoni* | predator |
| *Jasus edwardsii* | predator |
| *Jasus verreauxi* | predator |
| *Lethrinus miniatus* | predator |
| *Lethrinus olivaceus* | predator |
| *Lethrinus xanthochilus* | predator |
| *Lutjanus argentimaculatus* | predator |
| *Lutjanus bohar* | predator |
| *Lutjanus carponotatus* | predator |
| *Lutjanus monostigma* | predator |
| *Lutjanus rivulatus* | predator |

| | |
|---|---|
| *Lutjanus russellii* | predator |
| *Lutjanus sebae* | predator |
| *Lutjanus semicinctus* | predator |
| *Lutjanus vitta* | predator |
| *Myliobatis australis* | predator |
| *Pentaceropsis recurvirostris* | predator |
| *Plectropomus areolatus* | predator |
| *Plectropomus laevis* | predator |
| *Plectropomus leopardus* | predator |
| *Plectropomus maculatus* | predator |
| *Scorpaena papillosa* | predator |
| *Trygonorrhina fasciata* | predator |
| *Urolophus cruciatus* | predator |
| *Variola albimarginata* | predator |
| *Variola louti* | predator |
| *Acanthurus auranticavus* | herbivore |
| *Acanthurus bariene* | herbivore |
| *Acanthurus blochii* | herbivore |
| *Acanthurus dussumieri* | herbivore |
| *Acanthurus grammoptilus* | herbivore |

| | |
|---|---|
| *Acanthurus lineatus* | herbivore |
| *Acanthurus maculiceps* | herbivore |
| *Acanthurus nigricans* | herbivore |
| *Acanthurus nigricauda* | herbivore |
| *Acanthurus nigrofuscus* | herbivore |
| *Acanthurus olivaceus* | herbivore |
| *Acanthurus pyroferus* | herbivore |
| *Acanthurus triostegus* | herbivore |
| *Acanthurus xanthopterus* | herbivore |
| *Amblypneustes elevatus* | herbivore |
| *Amblypneustes ovum* | herbivore |
| *Amblypneustes* sp. (Cape Portland) | herbivore |
| *Amblypneustes* spp. | herbivore |
| *Bolbometopon muricatum* | herbivore |
| *Calotomus carolinus* | herbivore |
| *Centrostephanus rodgersii* | herbivore |
| *Cetoscarus bicolor* | herbivore |
| *Chlorurus bleekeri* | herbivore |
| *Chlorurus frontalis* | herbivore |
| *Chlorurus japanensis* | herbivore |

| | |
|---|---|
| *Chlorurus microrhinos* | herbivore |
| *Chlorurus sordidus* | herbivore |
| *Ctenochaetus* spp. | herbivore |
| *Dischistodus melanotus* | herbivore |
| *Dischistodus perspicillatus* | herbivore |
| *Dischistodus prosopotaenia* | herbivore |
| *Dischistodus pseudochrysopoecilus* | herbivore |
| *Echinometra mathaei* | herbivore |
| *Goniocidaris tubaria* | herbivore |
| *Haliotis laevigata* | herbivore |
| *Haliotis rubra* | herbivore |
| *Haliotis scalaris* | herbivore |
| *Heliocidaris erythrogramma* | herbivore |
| *Heliocidaris tuberculata* | herbivore |
| *Hemiglyphidodon plagiometopon* | herbivore |
| *Hipposcarus longiceps* | herbivore |
| *Holopneustes inflatus* | herbivore |
| *Holopneustes porosissimus* | herbivore |
| *Holopneustes purpurascens* | herbivore |
| *Holopneustes* spp. | herbivore |

| | |
|---|---|
| *Naso lituratus* | herbivore |
| *Naso tuberosus* | herbivore |
| *Naso unicornis* | herbivore |
| *Neoglyphidodon nigroris* | herbivore |
| *Phyllacanthus parvispinus* | herbivore |
| *Plectroglyphidodon dickii* | herbivore |
| *Plectroglyphidodon johnstonianus* | herbivore |
| *Plectroglyphidodon lacrymatus* | herbivore |
| *Pomacentridae* spp. | herbivore |
| *Pomacentrus adelus* | herbivore |
| *Pomacentrus chrysurus* | herbivore |
| *Pomacentrus grammorhynchus* | herbivore |
| *Pomacentrus nigromarginatus* | herbivore |
| *Pomacentrus wardi* | herbivore |
| *Pseudoboletia indiana* | herbivore |
| *Scarus altipinnis* | herbivore |
| *Scarus chameleon* | herbivore |
| *Scarus dimidiatus* | herbivore |
| *Scarus flavipectoralis* | herbivore |
| *Scarus forsteni* | herbivore |

| | |
|---|---|
| *Scarus frenatus* | herbivore |
| *Scarus ghobban* | herbivore |
| *Scarus globiceps* | herbivore |
| *Scarus longipinnis* | herbivore |
| *Scarus niger* | herbivore |
| *Scarus oviceps* | herbivore |
| *Scarus psittacus* | herbivore |
| *Scarus rivulatus* | herbivore |
| *Scarus rubroviolaceus* | herbivore |
| *Scarus schlegeli* | herbivore |
| *Scarus spinus* | herbivore |
| *Siganus argenteus* | herbivore |
| *Siganus corallinus* | herbivore |
| *Siganus doliatus* | herbivore |
| *Siganus javus* | herbivore |
| *Siganus lineatus* | herbivore |
| *Siganus puellus* | herbivore |
| *Siganus punctatissimus* | herbivore |
| *Siganus punctatus* | herbivore |
| *Siganus spinus* | herbivore |

| | |
|---|---|
| *Siganus vulpinus* | herbivore |
| *Stegastes apicalis* | herbivore |
| *Stegastes fasciolatus* | herbivore |
| *Stegastes gascoynei* | herbivore |
| *Stegastes nigricans* | herbivore |
| *Tripneustes gratilla* | herbivore |
| *Turbo torquatus* | herbivore |
| *Turbo undulatus* | herbivore |
| *Zebrasoma scopas* | herbivore |
| *Zebrasoma veliferum* | herbivore |
